# Unexpected contribution of the Fak system and the thioesterase TesE to the growth and membrane physiology of *Enterococcus faecalis*

**DOI:** 10.1128/jb.00121-25

**Published:** 2025-06-30

**Authors:** R. D. Johnston, T. A. Getty, B. M. Woodall, S. Maharjan, N. L. Arnold, W. B. Seaton, M. Stevenson, S. R. Campagna, E. M. Fozo

**Affiliations:** 1 Department of Microbiology, University of Tennessee 189504 , Knoxville, Tennessee, USA; 2 Department of Chemistry, University of Tennessee 312344 , Knoxville, Tennessee, USA; 3 Oaklahoma Baptist University, Shawnee, Oklahoma, USA; 4 Biological and Small Molecule Mass Spectrometry Core, University of Tennessee 4292, Knoxville, Tennessee, USA; Queen Mary University of London, London, United Kingdom

**Keywords:** FakB, Fak system, *Enterococcus faecalis*, TesE, thioesterase

## Abstract

*Enterococcus faecalis* is a native of the intestine and a hospital-acquired pathogen that uses host fatty acids to form its membrane. We investigated the utilization of exogenous fatty acids via the fatty acid kinase (Fak) system to understand the varied impacts fatty acids have on physiology. FakB proteins bind fatty acids, and FakA then phosphorylates them for lipid synthesis. Network analysis indicated that two of the four FakB proteins of *E. faecalis* OG1RF cluster with described proteins of *Staphylococcus aureus* and *Streptococcus pneumoniae* (FakB1, FakB2). However, two additional *E. faecalis* FakB proteins clustered separately and distinctly from characterized proteins; these were subsequently denoted as FakB4 and FakB5. A strain deleted for three of the four genes (*ΔfakB1,2,5* strain) had severe morphological defects when grown in rich media. Deletion of all four *fakB*-encoding genes was not possible unless a thioesterase encoding gene, *tesE*, was also deleted (*Δquint* strain). The *Δquint* strain behaved similarly to wild-type OG1RF in rich media, indicating that the combination of free fatty acids from the growth environment and those liberated via TesE was detrimental to the *ΔfakB1,2,5* strain. The *Δquint* strain grew unimpeded in saturated fatty acids that are normally toxic to *E. faecalis,* indicating that incorporation of these fatty acids into phospholipids mediates their toxicity. While saturated fatty acids reduced the membrane fluidity of wild-type OG1RF, they had no impact on the *Δquint* strain. Our combined data support that the Fak system in *E. faecalis* plays a critical role in maintaining membrane fluidity and driving enterococcal physiology.

**IMPORTANCE:**

Bacteria living within humans encounter a variety of fatty acids that they can use to synthesize their own cellular material. However, different fatty acids can have a variety of effects on the same bacterial species. Within, we examined how *Enterococcus faecalis*, which naturally lives in human intestines but can also cause disease, uses fatty acids from its environment. We discovered unexpectedly that fatty acid binding proteins contribute to many aspects controlling bacterial growth, shape, and behavior.

## INTRODUCTION

Many bacterial species have a range of interactions with their hosts. These bacteria can adapt to different host environments and may, in the same host, occupy both a commensal and a pathogenic niche. One such organism, *Enterococcus faecalis*, commensally colonizes the human gastrointestinal tract, yet upon entering wounds or other tissues, it can cause disease. *E. faecalis* is consequently exposed to fatty acid-rich host fluids like bile (gastrointestinal tract; commensal state) and serum (host tissues; pathogenic state), which impact its physiology (1). While *E. faecalis* cannot consume fatty acids as a carbon source (i.e., does not perform β-oxidation), it will use environmental fatty acids in lipid synthesis and to control gene expression (2–7).

**Fig 1:**
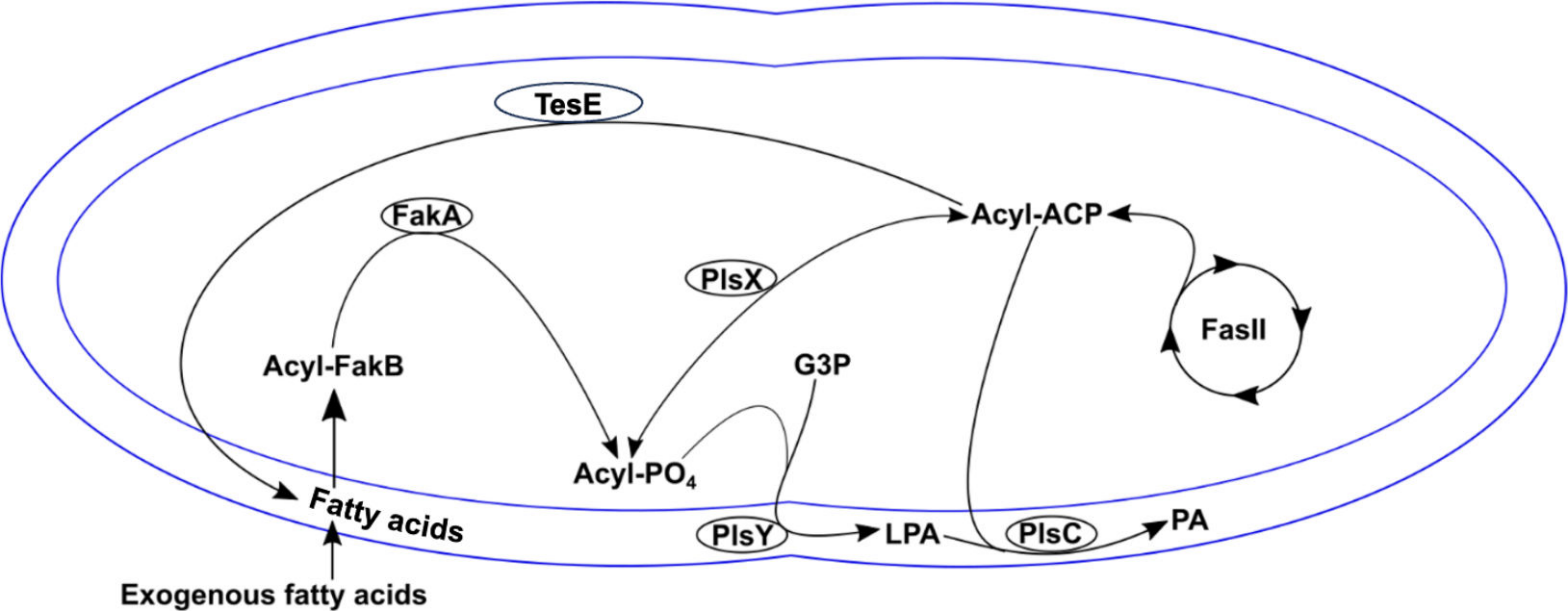
Synthesis and use of free fatty acids in *E. faecalis*. Free fatty acids, whether exogenously supplied or produced by cleavage of a fatty acid from acyl-ACP by TesE, are bound by a FakB protein. The FakA enzyme phosphorylates the fatty acid, forming acyl-phosphate (Acyl-PO4). Acyl-phosphates are incorporated onto the *sn-*1 position of glycerol-3-phosphate by PlsY to form lysophosphatidic acid (LPA). Acyl-phosphates can also be bound to ACP through the action of PlsX. Acyl-ACP, generated through the *de novo* fatty acid synthesis cycle (FasII), is used as the fatty acid donor to form phosphatidic acid, the precursor to membrane phospholipids.

Like *E. faecalis*, *Streptococcus pneumoniae* and *Staphylococcus aureus* do not perform β-oxidation, but they possess a fatty acid kinase (Fak) system to activate exogenous fatty acids for phospholipid synthesis (Fig. 1). FakB proteins bind fatty acids, which are then phosphorylated in conjunction with FakA (8, 9). The phosphorylated (“activated”) fatty acid is then incorporated onto glycerol-3-phosphate by the enzyme PlsY (forming lysophosphatidic acid) or attached to an acyl carrier protein (ACP) by the enzyme PlsX (9). PlsC then transfers a fatty acid from ACP onto lysophosphatidic acid, forming phosphatidic acid, which serves as a precursor to form phospholipids (9). In both species, FakB1 is thought to preferentially bind saturated fatty acids, and FakB2 unsaturated fatty acids; *S. pneumoniae* also possesses FakB3, which has specificity for polyunsaturated fatty acids (8–10). While their specificities remain unclear, we and others have noted that there are four FakB homologs in *E. faecalis* (7, 11).

Acyl-phosphates can also (Fig. 1) be produced by the cleavage of a fatty acid from acyl-ACP by the thioesterase TesE in *E. faecalis* and TesS in *S. pneumoniae* (12, 13). The newly generated free fatty acid can then be used as a substrate for the Fak system to generate acyl-phosphates (12). However, given that deletion of the *fak* genes in *S. aureus* and *S. pneumoniae* did not negatively impact the growth of the cells in routine laboratory media, this route is thought to be a minor pathway.

While *E. faecalis* can use exogenous fatty acids for lipid synthesis, the addition of an individual fatty acid can have varied effects on the organism. Growth with specific saturated fatty acids, such as palmitic (C_16:0_) or myristic (C_14:0_) acids, distorted cellular morphology, severely impacted generation time, and in some instances, caused a premature growth stasis (1, 14). On the contrary, the addition of either eukaryotic-derived oleic (C_18:1 *cis* 9_) or linoleic (C_18:2 *cis* 9,12_) acids, both prevalent in host fluids, allowed for growth in the presence of a *de novo* fatty acid biosynthesis inhibitor and induced tolerance to membrane-damaging agents (14, 15). Despite the vast differences in physiological outcome, both “toxic” fatty acids (e.g., palmitic acid) and “nontoxic” fatty acids (e.g., oleic acid) are found in bile and serum (1).

Why certain fatty acids are toxic or nontoxic to *E. faecalis* is not known. As fatty acids can be detected free in the membrane (i.e., not attached to a lipid headgroup) or as part of a phospholipid, we wondered if accumulation in either case was driving the observed effects (3–5). We thus attempted to delete the *fak* genes from *E. faecalis* OG1RF to determine their contributions to fatty acid-induced toxicity. Unexpectedly, we discovered that the loss of three of the four *fakB* encoding genes of *E. faecalis* impaired growth in rich media. We were only able to delete all four *fakB* genes when combined with the deletion of *tesE*. We confirmed that this quintuple deletion strain (*ΔfakB1,2,4,5/ΔtesE,* referred to as the *Δquint* strain) was dependent upon *de novo* fatty acid biosynthesis for membrane formation, as the addition of exogenous fatty acids could not overcome growth inhibition by cerulenin. While the addition of specific saturated fatty acids inhibited the growth of the wild-type strain, these same fatty acids had no effect on the growth of the *Δquint* strain, suggesting that incorporation of these fatty acids into phospholipids is responsible for their toxicity. The addition of exogenous saturated fatty acids also had little to no impact on the membrane fluidity of the *Δquint* strain, suggesting that head group placement of fatty acids is the major driver for membrane fluidity and that a functional Fak system contributes to membrane physiology.

## RESULTS

### Identification of *Enterococcus faecalis* FakA and FakB homologs

Previous work demonstrated that the growth of *E. faecalis* OG1RF (OG1RF herein) in exogenous fatty acids altered both fatty acid tails and polar head group composition of membrane lipids, resulting in significant physiological changes (1, 3–5, 7, 13–15). We wanted to conclude if OG1RF utilizes the FakA/B system as described in *S. aureus* and *S. pneumoniae*, with FakB proteins binding fatty acids via a DegV domain, followed by phosphorylation of the fatty acid via FakA, activating them for lipid synthesis (8, 9). Other groups have noted four possible FakB homologs in *E. faecalis* strains (7, 11). We identified within OG1RF a single homolog of FakA (OG1RF_RS12165) and four homologs of the FakB proteins of *S. aureus* and *S. pneumoniae* (OG1RF_RS00080, OG1RF_RS07200, OG1RF_RS05020, and OG1RF_RS06660).

We compared the OG1RF FakB proteins to those of *S. aureus* and *S. pneumoniae* in an attempt to predict the specificity of the enterococcal homologs (Table S1). However, the protein identities and positive substitutions did not lead to any clear conclusions as to potential specificity. Predicted protein structures and known residues conserved for fatty acid binding also did not aid in predicting the fatty acid specificity for each OG1RF FakB protein (Fig. S1 and S2) (16, 17). Thus, we opted to use network analysis to better decipher potential fatty acid specificity.

### Network analysis of DegV domains reveals multiple FakB proteins across Gram-positive species

**Fig 2:**
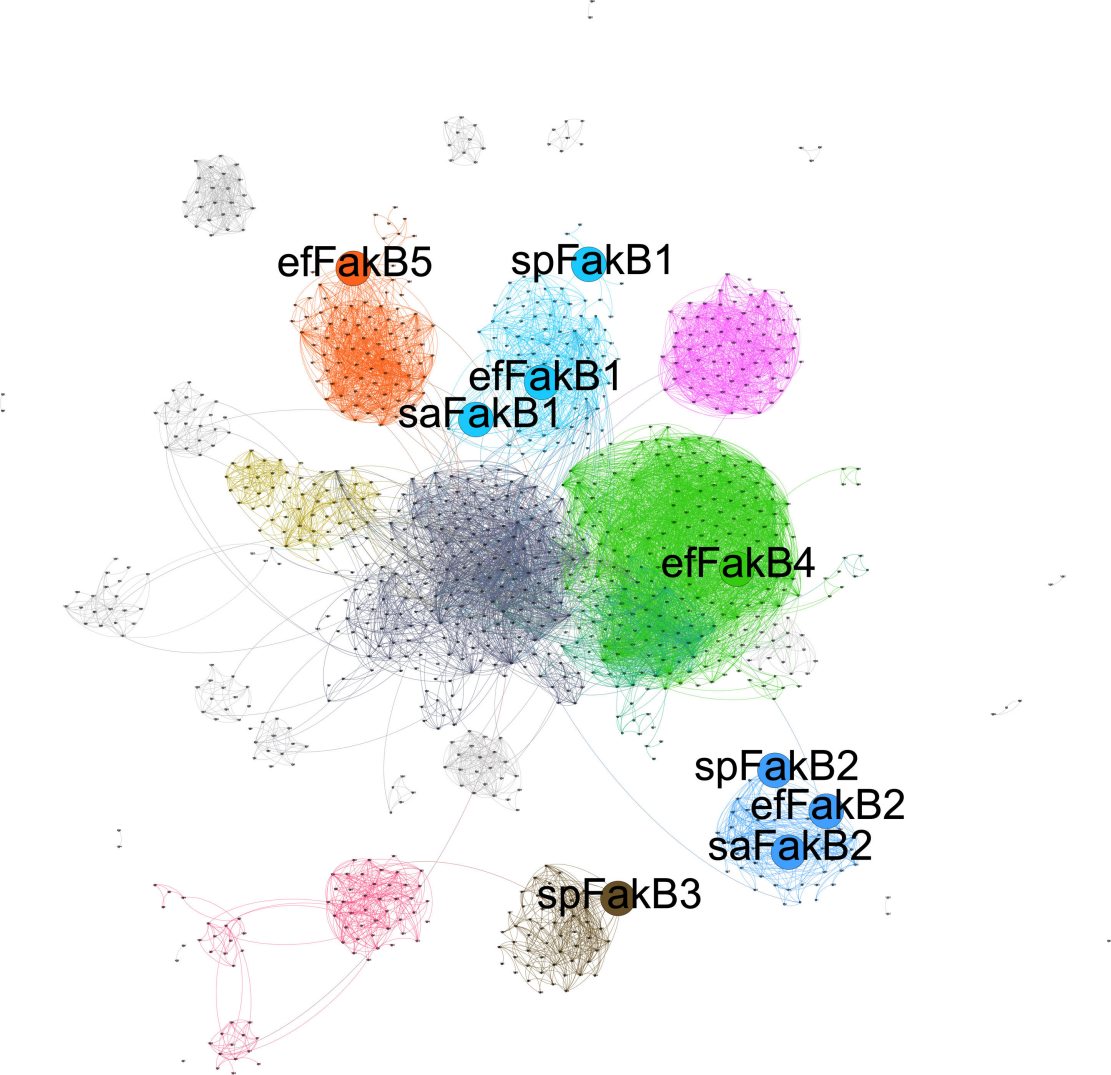
*E. faecalis* FakB homologs cluster distinctly. SSN of DegV protein domains (protein family = PF02645) generated by the Enzyme Function Initiative’s Enzyme Similarity Tool (EFI-EST). Each node is representative of a group of proteins sharing ≥50% identity over 80% of the sequence (see Materials and Methods) with each other; although in many cases, they are from different organisms. Edges were drawn between nodes using an alignment score of 55, resulting in edges being drawn only between nodes where the DegV domains represented therein had ≥35% identity. 641B1. The darker the color, the higher the network connectivity. Diagram presents network clustering of DegV sequences, with different communities colored by network modularity. The nodes representing the FakB proteins of *S. aureus*, *S. pneumoniae,* and *E. faecalis* are enlarged within the plot.

We generated a sequence similarity network (SSN) of DegV domains (Pfam = PF02645) and annotated the locations of the FakB homologs of *E. faecalis* along with those of *S. aureus* and *S. pneumoniae* (Fig. 2). We found that OG1RF_RS06660 clustered with saFakB1 (*S. aureus* FakB1) and spFakB1 (*S. pneumoniae* FakB1), which are associated with saturated fatty acid binding (8, 9). OG1RF_RS07200 clustered with saFakB2 and spFakB2, indicating similarity with proteins that bind monounsaturated fatty acids. We thus named OG1RF_RS06660 and OG1RF_RS07200 FakB1 and FakB2, respectively. None of the *E. faecalis* FakB homologs clustered with spFakB3. OG1RF_RS00080 was located in a highly connected portion of the network, yet did not cluster with annotated FakB proteins, so we named it FakB4. Similarly, OG1RF_RS05020 clustered alone in a separate branch of the network and was designated as FakB5.

Given the number of FakB proteins varied between *E. faecalis*, *S. aureus,* and *S. pneumonia*e, we wondered whether the number of FakB homologs was species-specific. 95% of *S. aureus* strains had two DegV domains within separate nodes (two unique FakB proteins, Fig. S3), while 87% of *S. pneumoniae* isolates appeared in three nodes (87% of isolates), indicating the presence of three DegV domains within species (3 FakB proteins, Fig. S3). However, *E. faecalis* isolates predominantly had 4 DegV domain-containing proteins, as isolates appeared in four nodes (96% of isolates, four FakB proteins, Fig. S3).

Overall, we noted that for our clustering analyses, the DegV domains identified were predominantly found in Gram-positive isolates, with numerous intestinal isolates clustering with efFakB4 and efFakB5 (Fig. S4).

### Combined deletion of *fakB* genes impacts growth in specific fatty acids

Past studies demonstrated that the addition of individual fatty acids within growth media had a variety of impacts on OG1RF. While the addition of oleic acid (C_18:1 cis 9_) had no impact on growth or morphology, supplementation with either saturated fatty acid palmitic (C_16:0_), myristic (C_14:0_), or stearic (C_18:0_) acids increased the generation time of OG1RF and distorted morphology (14, 15). The mechanism behind saturated fatty acid-induced toxicity, though, has remained unknown. In *S. aureus*, growth defects upon supplementation with unsaturated fatty acids are alleviated upon deletion of *fakA* or its two *fakB* genes (18). We hypothesized that the increased incorporation of saturated fatty acids onto lipid headgroups by FakB proteins in OG1RF may explain the toxic effects of saturated fatty acids. We deleted the individual *fakB* genes and monitored growth in the presence or absence of fatty acids. Strains with single *fakB* genes deleted were similar to the parental OG1RF when grown in the tested saturated and unsaturated fatty acids, with some exceptions (Table S2, data not shown). Combining gene deletions did result in notable growth differences in fatty acids—for instance, the *ΔfakB1,5* strain had long generation times in saturated fatty acids, while the *ΔfakB1,2* strain had particularly long generation times in unsaturated fatty acids (Table S2). However, we were unable to generate a quadruple *fakB* deletion strain unless *tesE* was also deleted (see below).

**Fig 3:**
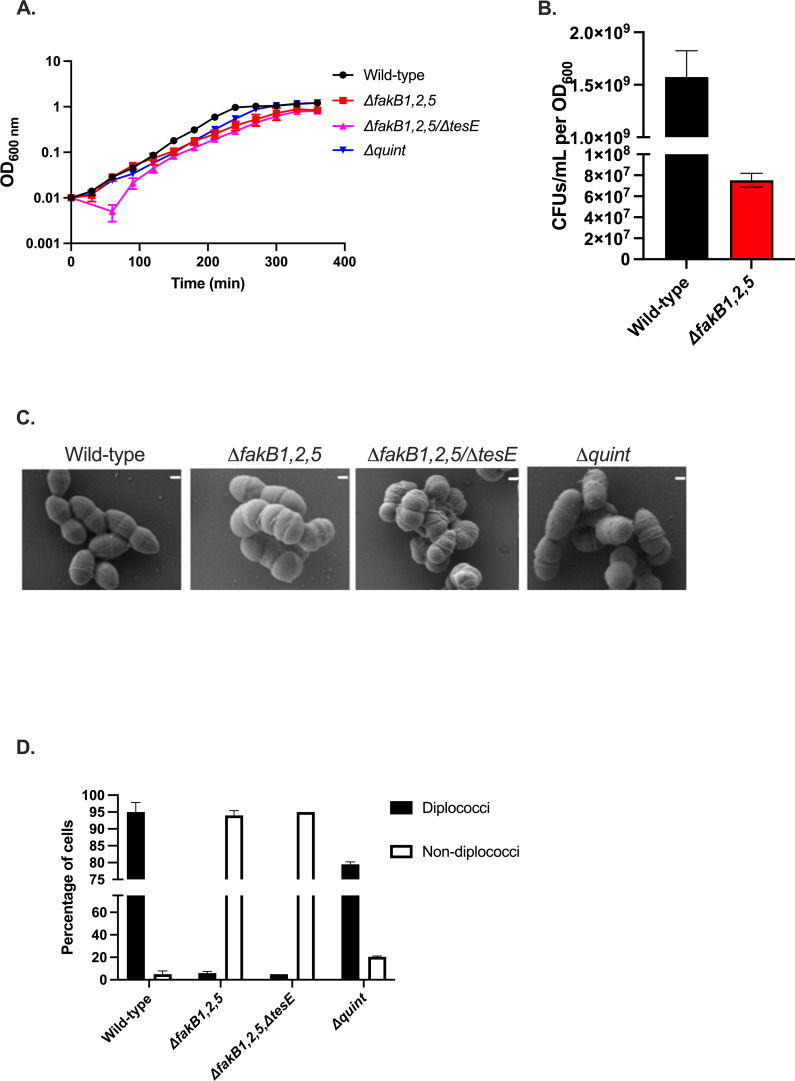
*ΔfakB1,2,5* strain has severely distorted morphology when grown in rich media (BHI). Growth of wild-type *E. faecalis,* Δ*fakB1,2,5, ΔfakB1,2,5/∆tesE,* and *∆quint* strains in plain BHI, shown are averages ± standard deviations of *n* = 3 (**A**); colony forming units of wild-type *E. faecalis* and Δ*fakB1,2,5* strains normalized to OD_600 nm_, shown are averages and standard deviations of *n* = 3; *P* = 0.0014 as determined by Welch’s *t*-test (**B**); SEM of wild-type *E. faecalis, ΔfakB1,2,5, ∆fakB1,2,5/tesE* and *∆quint* strains grown to mid-logarithmic phase in BHI. Scale bar is 200 nm (**C**); average percentages of diplococci vs non-diplococci ± standard deviations for a total of *n* = 100 cells across 5–7 images per biological replicate (D).

To the *ΔfakB1,2* strain, we deleted *fakB5,* and this resulting strain, *ΔfakB1,2,5*, had an increased generation time in rich media (brain heart infusion; BHI) without supplements compared to the wild-type strain (Fig. 3A; Table S2). For a given OD_600_ value, the *ΔfakB1,2,5* strain had approximately 10-fold fewer CFUs relative to wild type when grown in BHI (Fig. 3B; *P* = 0.0014). Given this and that *ΔfakB1,2,5* cells tended to clump at the bottom of the tube after overnight growth (data not shown), we examined cellular morphology via scanning electron microscopy (SEM). As shown in Fig. 3C, wild-type OG1RF possessed an elongated diplococcus morphology when grown in BHI. However, *ΔfakB1,2,5* cells were very irregular in shape and size, with clear hyperseptation relative to the wild-type control (Fig. 3C). Indeed, only a small percentage of the *ΔfakB1,2,5* cells were diplococci (Fig. 3D, 5%).

Like its parental strain, *ΔfakB1,2,* the *ΔfakB1,2,5* strain had a significantly longer generation time than that of wild-type OG1RF (92.6 vs 37.9 min, *P* < 0.01) in oleic acid (C_18:1 *cis* 9_) as well as linoleic acid (C_18:2 *cis* 9,12_; 135.3 vs 66 min, *P* < 0.01; Table S2; see Discussion). On the contrary, growth of the *ΔfakB1,2,5* strain in stearic acid (C_18:0_) actually had a decreased generation time in comparison to a wild-type strain (60.3 vs 82 min, *P* < 0.02), suggestive of reduced toxicity (see below, Table S2).

### Combined deletion of *fakB1*, *fakB2*, and *fakB5* leads to an increase in saturated:unsaturated fatty acids

The morphology of the *ΔfakB1,2,5* strain in rich media (BHI) was reminiscent of wild-type OG1RF cells grown in the presence of the saturated fatty acid palmitic acid (C_16:0_) or myristic acid (C_14:0_). In either case, the supplied saturated fatty acid dominated the membrane profile (14). We performed gas chromatography fatty acid methyl ester (GC-FAME) analysis to conclude whether the *ΔfakB1,2,5* strain had increased levels of saturated fatty acids compared to the wild-type strain when grown in BHI (Table 1). Note that this analysis detects all fatty acids present regardless of being attached to phospholipid head groups or being free. We observed a ~7% increase in palmitic acid (C_16:0_) in the *ΔfakB1,2,5* strain (*P* = 0.002), combined with a ~14% decrease in *cis*-vaccenic acid (C_18:1 *cis* 11_; *P <* 0.001, Table 1). Overall, the *ΔfakB1,2,5* strain had a ratio of saturated fatty acids to unsaturated fatty acids of 2:1 vs a 1:1 in wild type (Table 1).

**TABLE 1:** GC-FAME analysis of strains during log phase growth in BHI^*b*^

Fatty acid	Wild type	*ΔfakB1,2,5*	*Δquint*
C_12:0_	0.9 ± 0.0	0.9 ± 0.1	0.1 ± 0.2
C_14:0_	4.3 ± 0.1	5.4 ± 0.1	5.5 ± 0.0
C_16:1_	5.7 ± 0.0	4.8 ± 0.1	6.6 ± 0.1
C_16:0_	41.4 ± 0.7	48.4 ± 0.1	37.2 ± 0.7
C_17:1 *cis10*_	1.0 ± 0.8	3.1 ± 0.1	2.3 ± 0.2
C_17:0_ 2OH	4.4 ± 0.1	5.8 ± 0.1	6.1 ± 0.2
C_18:1 *cis11*_	37.0 ± 0.5	22.7 ± 0.4	37.0 ± 0.5
C_18:0_	4.8 ± 0.1	3.6 ± 0.2	3.5 ± 0.0
C_19:0 cyclo11_	0.6 ± 0.0	1.6 ± 0.1	1.4 ± 0.2
Unknown	ND	1.9 ± 0.6	0.2 ± 0.4
Others^*a*^	ND	0.9 ± 0.2	0.1 ± 0.1
Saturated/unsaturated ratio	1.3 ± 0.0	2.1 ± 0.1	1.2 ± 0.0

^*a*^ Others are fatty acids which comprised <1% of the total membrane content.

^*b*^ Shown are the averages ± standard deviations for *n* = 3 replicates. ND indicates that the fatty acid indicated was not detected for the given strain.

### Growth in defined media restores morphology of *ΔfakB1,2,5*

**Fig 4:**
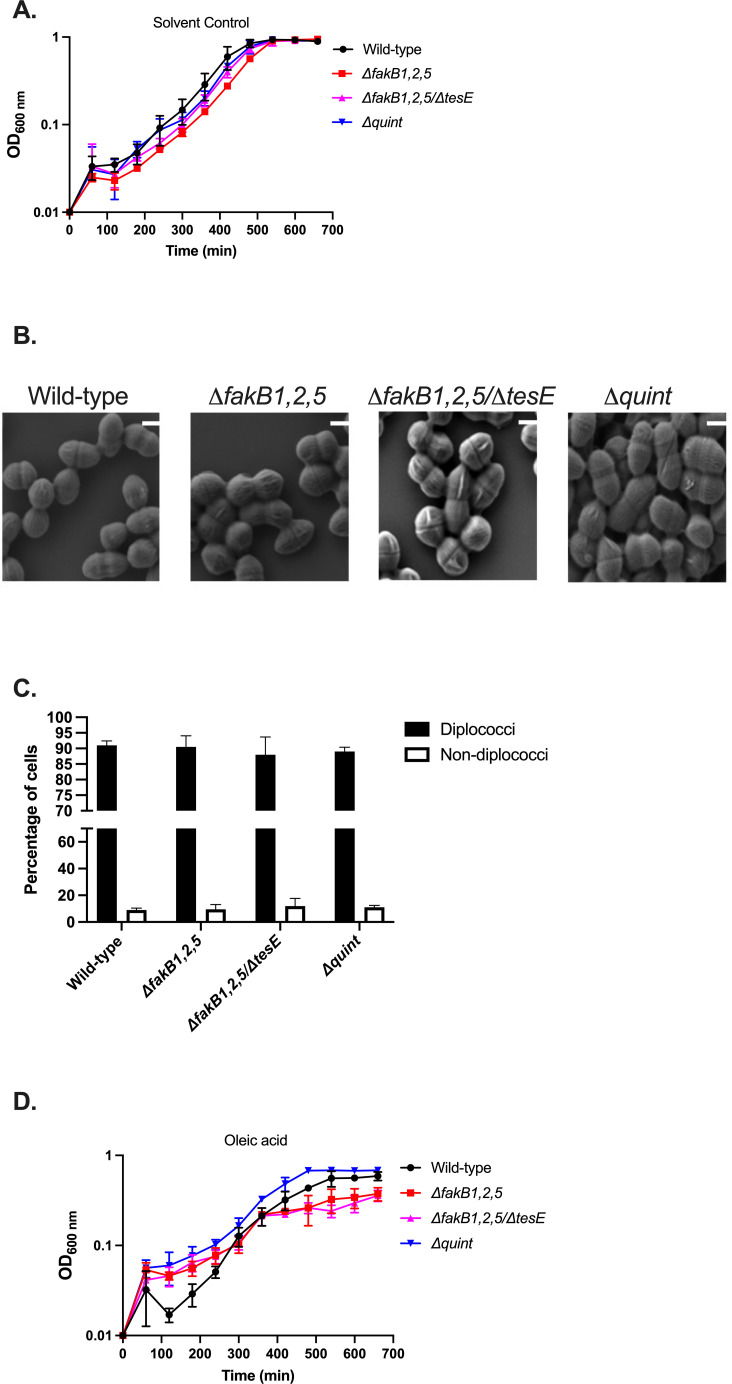
Growth in defined media reverts growth and morphological phenotypes for the *ΔfakB1,2,5* strain. Growth of wild-type *E. faecalis,* Δ*fakB1,2,5, ΔfakB1,2,5/∆tesE, and ∆quint* strains in CDM-sm with solvent control, shown are averages and standard deviations of *n* = 3 (**A**); SEM of wild-type *E. faecalis, ΔfakB1,2,5 ∆fakB1,2,5/∆tes*E, and *∆quint* strains grown to mid-logarithmic phase in CDM-sm. Scale bar is 200 nm (**B**); average percentages of diplococci vs non-diplococci ± standard deviations for a total of *n* = 100 cells across 5–7 images per biological replicate (**C**); growth of wild-type *E. faecalis,* Δ*fakB1,2,5, ΔfakB1,2,5/∆tesE,* and *∆quint* strains in CDM-sm with 20 mg/mL oleic acid, shown are averages and standard deviations of *n* = 3 (**D**).

Given the altered fatty acid content and distorted morphology of the *ΔfakB1,2,5* strain in BHI, we hypothesized that trace saturated fatty acids in the media could be contributing to these observations. A recent study reported the detection of both palmitic acid (C_16:0_) and stearic acid (C_18:0_) within BHI (19). Similarly, we noted an increased generation time as well in tryptic soy broth (TSB, Fig. S5), which has also been shown to contain the same saturated fatty acids (20). We thus examined morphology and growth within a defined medium (chemically defined minimal-sm [CDM-sm], Table S4) of the *ΔfakB1,2,5* strain (Table S3). As shown in Fig. 4A, the deletion strain grew similarly to the wild-type strain in defined media, and the majority of cells possessed typical diplococcal morphology (approximately 95%, Fig. 4B and C).

We noted above that the *ΔfakB1,2,5* strain had a significant increase in generation time when grown in rich media supplemented with oleic acid, while the wild-type strain grew unimpeded. We thus examined the growth of the strain in CDM-sm with oleic acid to determine if the effects would phenocopy what was observed in rich media with the fatty acid. The addition of oleic acid (C_18:1 *cis*9_) greatly reduced the generation time of the *ΔfakB1,2,5* strain, as was seen when oleic acid was added to rich media (Table S2). Surprisingly, even the wild-type strain grew far slower in defined media supplemented with oleic acid compared to solvent control (Fig. 4D, see Discussion).

### Deletion of *tesE* allows for the deletion of all four *fakB* genes in rich media

As the observed phenotypes of the *ΔfakB1,2,5* strain were dependent upon the presence of fatty acids in the growth environment, we hypothesized that the activity of TesE may exacerbate these phenotypes in rich media. TesE is a thioesterase that cleaves fatty acids from the acyl carrier protein (13). The resultant free fatty acids are bound and phosphorylated via the Fak system and then enter lipid synthesis or contribute to the repression of *de novo* fatty acid biosynthesis (Fig. 1) (7, 12). We hypothesized that loss of multiple *fakB* genes could result in free fatty acid buildup due to continued activity of TesE, resulting in the altered lipid composition of the *ΔfakB1,2,5* strain and potential detrimental growth effects in BHI (see Discussion). We predicted that upon deletion of *tesE*, we would be able to delete the final *fakB* gene and potentially revert the severe phenotypes observed. We deleted *tesE* in the *ΔfakB1,2,5* strain, generating the *ΔfakB1,2,5/ΔtesE* strain. We were then able to delete all *fakB* genes, generating the *ΔfakB1,2,4,5/ΔtesE* strain (referred to as the *Δquint* strain herein).

The *Δquint* strain grew similarly to wild-type OG1RF in BHI with diplococcal morphology, unlike the severely distorted morphologies of the *ΔfakB1,2,5* strain and the *ΔfakB1,2,5/ΔtesE* strain (Table S2; Fig. 3A, C, and D; see Discussion). Notably, some of the fatty acid alterations observed in the *ΔfakB1,2,5* strain were reversed in the *Δquint* strain (Table 1). The *Δquint* strain had a significant reduction in palmitic acid (C_16:0_) and a significant increase in *cis*-vaccenic acid (C_18:1 *cis11*_) relative to the *ΔfakB1,2,5* strain.

**Fig 5:**
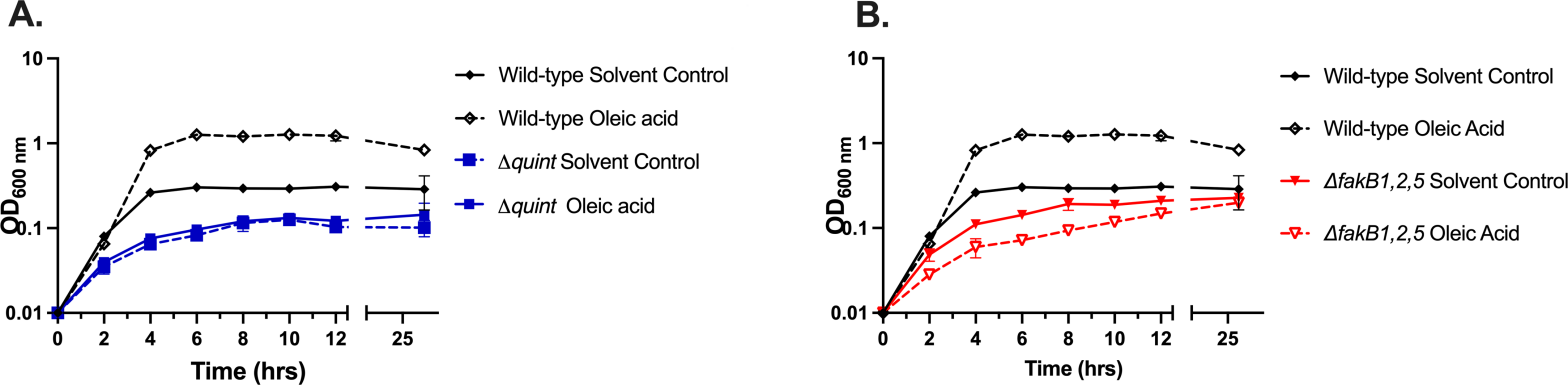
Oleic acid does not rescue growth inhibition by cerulenin in the *Δquint* and *ΔfakB1,2,5* deletion strains. Wild-type *E. faecalis* and Δ*quint* strains (**A**) and wild-type *E. faecalis* and Δ*fakB1,2,5* strains (**B**) were grown in BHI containing 5 µg/mL cerulenin. The cultures were supplemented with 5 µg/mL oleic acid or an equivalent volume of solvent as indicated (see Materials and Methods for full details). Represented are average OD_600_ values ± standard deviations for *n* = 3 replicates.

Wild-type *E. faecalis* can overcome inhibition of *de novo* fatty acid biosynthesis by the antibiotic cerulenin, if supplied with specific fatty acid(s) in its growth media (14, 15). This is due to the Fak system generating acyl-phosphates required for lipid synthesis and the interconversion of acyl-phosphate to acyl-ACP by PlsX (Fig. 1). To confirm that the *Δquint* strain could no longer utilize exogenous fatty acids to support lipid synthesis, we examined its growth in the presence of cerulenin and with or without the addition of oleic acid. Like the wild-type strain, growth was inhibited by cerulenin (Fig. 5A). However, whereas the addition of oleic acid (C_18:1 *cis* 9_) allowed for growth in the presence of cerulenin for wild-type OG1RF, it could not for the *Δquint* strain (Fig. 5A). The inherent toxicity of oleic acid to the *ΔfakB1,2,5* strain was even observed in the presence of cerulenin, suggestive of additional regulatory/metabolic problems for this strain (Fig. 5B).

To complement our data, we monitored the incorporation of ^13^C-labeled fatty acids into the lipidome in our wild type, *ΔfakB1,2,5,* and *Δquint* strains (4). Within 30 min after the addition of ^13^C oleic acid, we detected placement of the fatty acid onto lipid head groups for the wild type and *ΔfakB1,2,5* strains, but not for the *Δquint* strain; oleic acid was only found free within the *Δquint* strain (Table S5). We also examined ^13^C palmitic incorporation as well: similar results were found upon 30 min exposure of ^13^C palmitic acid, with no detection of the labeled fatty acid on lipid headgroups for the *Δquint* strain (Table S6). When grown with reduced levels of palmitic acid (see below, 2.5 mg/mL) until mid-exponential phase, the vast majority of ^13^C palmitic acid was found free in the *Δquint* strain (Table S6). Combined with the data above, the *Δquint* strain lacks a functional Fak system.

### Deletion of *tesE* and all *fakB* genes enables growth in toxic saturated fatty acids

**Fig 6:**
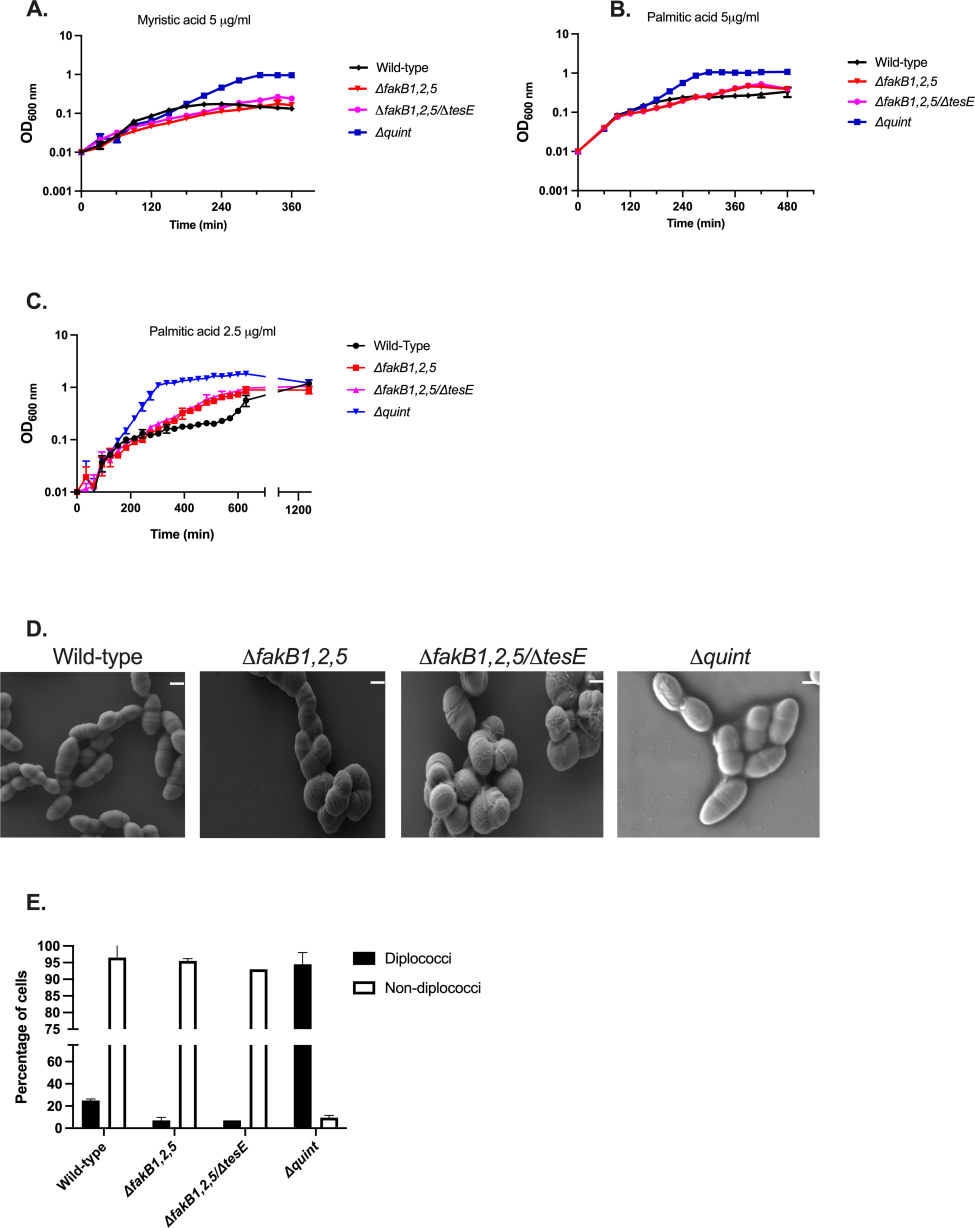
The Δ*quint* strain grows unimpeded in saturated fatty acids. Growth of wild-type *E. faecalis,* Δ*fakB1,2,5, ΔfakB1,2,5/∆tesE,* and *∆quint* strains in BHI supplemented with 5 µg/mL myristic acid (**A**); 5 µg/mL palmitic acid (**B**); 2.5 µg/mL palmitic acid (**C**), shown are the averages ± standard deviations for *n* = 3. SEM of wild-type *E. faecalis, ΔfakB1,2,5, ∆fakB1,2,5/∆tes*E, and *∆quint* strains grown in BHI and then exposed to 5 µg/mL palmitic acid for 30 min. Scale bar is 300 nm (**D**); average percentages of diplococci vs non-diplococci ± standard deviations for a total of *n* = 100 cells across 5–7 images per biological replicate (**E**).

The saturated fatty acids myristic acid (C_14:0_) and palmitic acid (C_16:0_) negatively impact cellular growth and result in severely distorted morphology of *E. faecalis* (14, 15). Growth in either fatty acid, when supplemented at 5 µg/mL, occurred only upon outgrowth of a genetic suppressor (mutant) cell (14). We asked whether the incorporation of exogenous saturated fatty acids onto lipid groups by the Fak system is responsible for these negative effects. The *Δquint* strain grew with a generation time of approximately 40–45 min in media containing either palmitic acid or myristic acid, while the other strains were inhibited at 5 µg/mL (Table S2; Fig. 6A and B). We noted that when grown in 2.5 µg/mL palmitic acid (half the concentration used for Fig. 6A), the *ΔfakB1,2,5* and Δ*fakB1,2,5/ΔtesE* grew, though not as robustly as compared to the *Δquint* strain (Fig. 6C).

Given the improved growth of the *Δquint* strain, we hypothesized that its morphology would be minimally impacted by saturated fatty acids. We grew strains in BHI until approximately mid-exponential phase and then exposed the cultures to 5 µg/mL palmitic acid for 30 min, followed by processing for SEM. As shown in Fig. 6D and E, the *Δquint* strain also maintained diplococcal morphology when grown in palmitic acid, unlike the wild-type strain. Thus, it is the placement of saturated fatty acids onto headgroups that drives their negative effects on *E. faecalis* physiology.

### Alteration of membrane fluidity by exogenous fatty acids is driven by a functional Fak system

**Fig 7:**
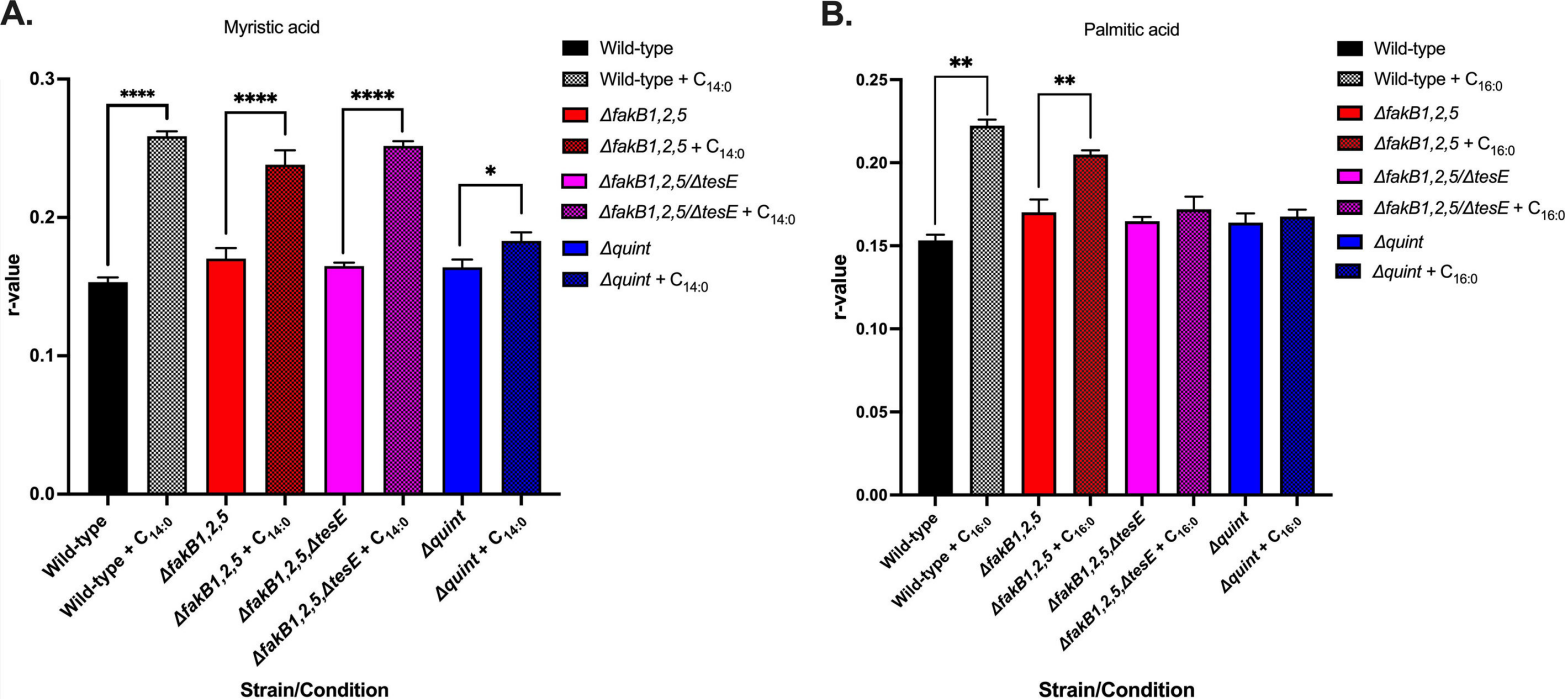
Saturated fatty acids have minimal impact on the fluidity of the *∆quint* strain. Wild-type *E. faecalis,* Δ*fakB1,2,5, ΔfakB1,2,5/∆tesE,* and *∆quint* strains were grown in BHI to mid-logarithmic phase and supplemented for 30 min with either solvent control, 5 µg/mL myristic acid (**A**) or 5 µg/mL palmitic acid (**B**), and anisotropy was performed. Shown are the average *r* values ± standard deviations for *n* = 3 replicates. An increase in *r* indicates a decrease in fluidity. ****P < 0.0001; **P = 0.0001–0.005 as determined by Welch’s *t*-test.

Supplying exogenous saturated fatty acids not only negatively impacts the growth and morphology of OG1RF but also results in decreased membrane fluidity as measured by the dye DPH (anisotropy) (15). Given that the combined loss of the *fakB* and *tesE* genes ameliorated the negative effects on growth by saturated fatty acids (Fig. 6; Table S2) and morphology (data not shown), we examined if their loss impacted membrane fluidity as well. The addition of myristic acid did result in an increased *r* value, i.e., reduced fluidity for all strains (Fig. 7A). However, the fold increase in the *r* value for the wild-type strain was 1.7, while for the *Δquint* strain, it was only 1.1. Palmitic acid addition only resulted in an increased *r* value for the wild-type strain (1.4-fold increase) and the *ΔfakB1,2,5* strain (1.2-fold, Fig. 7B). Neither the *ΔfakB1,2,5/ΔtesE* nor the *Δquint* strains had any changes in fluidity compared to their respective controls. Combined, the Fak system, along with TesE, contributes to the membrane fluidity of *E. faecalis* in fatty acid-rich environments.

## DISCUSSION

Within, we demonstrate that *E. faecalis* possesses a Fak system for the utilization of exogenous fatty acids and its subsequent contribution to membrane fluidity and bacterial growth. *E. faecalis* encodes four FakB proteins, two of which cluster independently of previously characterized homologs (Fig. 2). Deletion of individual *fakB* genes produced few (with some exceptions) growth phenotypes in rich media. Unexpectedly, a *ΔfakB1,2,5* strain had altered morphology and reduced growth in rich media, but these phenotypes were lost if grown in chemically defined media lacking fatty acids. Generation of a strain deleted for all *fakB* genes was only possible upon deletion of *tesE*, encoding a thioesterase that liberates fatty acids bound to ACP (Fig. 1). The resulting *Δquint* strain was more similar to the wild-type strain in comparison to *ΔfakB1,2,5* in morphology and cellular growth. This strain was able to grow in concentrations of myristic and palmitic acids that are normally toxic to *E. faecalis*. Furthermore, exogenously supplied saturated fatty acids did not alter bulk membrane fluidity of the *Δquint* strain, supporting that the Fak system contributes to membrane fluidity by activating fatty acids for placement on lipid headgroups, altering membrane composition. Thus, the Fak system is a major contributor to membrane fluidity.

The contribution of the Fak system in fatty acid toxicity has been examined in *S. aureus*; however, there are some notable differences between it and *E. faecalis. S. aureus* grows poorly in the polyunsaturated fatty acid linoleic acid (C_18:2 *cis 9*, 12_) and the monounsaturated fatty acid oleic acid (C_18:1 *cis* 9_). The deletion of *fakA* or the two *fakB*-encoding genes vastly improved the growth of *S. aureus* in either fatty acid (18). For *E. faecalis*, growth with the saturated fatty acids myristic (C_14:0_) and palmitic (C_16:0_) acids was particularly toxic: at 5 mg/mL (22 and 19 mM, respectively), growth occurred only upon selection of a genetic suppressor (14). The *Δquint* strain (Fig. 6; Table S2) grew unimpeded in either myristic acid or palmitic acid at these same concentrations. Regardless of whether one is examining the findings in *S. aureus* or *E. faecalis*, the combined data support that it is the activation of fatty acids for phospholipid assembly that results in detrimental phenotypes.

How, though, would this contribute to toxicity? It has been shown that in *E. faecalis*, exogenously supplied saturated fatty acids are far less effective at repressing *de novo* fatty acid biosynthesis than unsaturated fatty acids (in particular, oleic acid) (7). Palmitic acid is approximately 35%–40% of the total detected membrane fatty acids in *E. faecalis* when grown in rich media; addition of palmitic acid to rich media results in this single species comprising 70%–85% of the total detected fatty acids, with a subsequent reduction in the dominant unsaturated fatty acid, *cis*-vaccenic acid (C_18:1 *cis* 11_) (1, 13–15). This imbalance in membrane composition results in an overall reduction in membrane fluidity, which could then impact membrane protein interactions and activities, likely contributing to the toxic effects observed (15).

A similar imbalance in membrane composition may contribute to the observed growth and morphological differences in the *ΔfakB1,2,5* strain. Morphologically, this strain resembles wild-type OG1RF grown in saturated fatty acids; indeed, the *ΔfakB1,2,5* strain has increased levels of palmitic acid with substantially lower levels of detectable *cis*-vaccenic acid within its membrane (Table 1). As BHI media may contain palmitic acid, it is likely that the combination of trace saturated fatty acids in the media, along with continued *de novo* saturated fatty acid production, results in the altered membrane composition of this strain and subsequent growth/morphological issues (6, 7, 19). However, there are likely additional metabolic or gene regulatory mechanisms contributing to the alterations in the lipid profile. For example, the lipids labeled with ^13^C palmitic in the *ΔfakB1,2,5* strain were not as great as the number observed with the wild-type strain (Table S6). While this is reasonable given a loss of FakB proteins for activation of exogenous fatty acids, why is there such an elevated level of palmitic acid vs *cis*-vaccenic acid in this strain? Further analyses are needed to tease apart the impacts of the loss of FakB proteins on enterococcal membrane composition.

It was surprising to note how sensitive the *ΔfakB1,2,5* strain was to oleic acid compared to the wild-type strain (Tables S2 and S3) (1, 14, 15). The Cronan group has shown that oleic acid is very effective at repressing *de novo* fatty acid biosynthesis (7). For a strain with an intact Fak system, while oleic acid can repress *de novo* synthesis, it can also be effectively routed to form functional phospholipids; indeed, the addition of oleic acid allows the cells to overcome cerulenin inhibition (4, 14, 15). When supplemented with ^13^C oleic acid, the lipids of the *ΔfakB1,2,5* strain contained less labeled oleic acid when compared to wild type, supportive of the reduced activity of the Fak system. But why would this result in growth effects? One potential reason may be the consequence of the specific *fakB* genes deleted: although we noted that a *ΔfakB1,2,4* strain had an increased generation time when supplemented with oleic acid compared to the wild-type strain, it was not as severe as seen with the *ΔfakB1,2,5* strain (Table S2).

Currently, we lack data for the fatty acid binding preferences of enterococcal FakB proteins. The Cronan laboratory has been actively working in this area, but as of yet, has not reported biochemical specificity (7). Along with this, a FakB protein must interact with FakA for fatty acid activation. While recent work in *S. aureus* has demonstrated the interactions with saFakB2 and FakA, given four FakB proteins in *E. faecalis*, the affinity of an individual FakB protein with FakA could be variable (20, 21). Once the fatty acid is phosphorylated, FakB will interact with either PlsY or PlsX: again, it is possible that an individual FakB protein may have a different propensity for interacting with either acyltransferase. We also do not yet know if the expression of an individual *fakB* gene is equivalent to the others in *E. faecalis*. Such additional studies may help explain why a *ΔfakB1,2,5/ΔtesE* strain often phenocopies the *ΔfakB1,2,5* strain. Thus, more in-depth biochemical and regulatory analyses are needed to better ascertain these unexpected behaviors.

An important note regarding our findings: we were able to delete the *tesE* encoding gene in both our wild-type OG1RF and the *ΔfakB1,2,5* strains, which is in contradiction to a report from the Cronan group (13). In their work, they used *E. faecalis* F2-2; in examining the sequence region surrounding *tesE* for each strain, we noted no major differences that could explain why we were able to delete *tesE* in our background (data not shown). It is possible that OG1RF harbors another genetic difference(s) that allows for *tesE* to be deleted. We note there are two unique transposon insertions within *tesE* in the transposon library generated for OG1RF (22, 23). This would suggest that a pre-existing genetic difference exists between OG1RF and F2-2, but more work is needed.

This study also indicates the unique aspects of the Fak system across bacterial species. For *S. aureus* and *S. pneumoniae*, deletion of all *fakB* genes or *fakA* was possible, and for S*. aureus*, there were minor, if any, growth differences from parental strains in rich media (18). We were unable to delete all four *fakB* genes (unless *tesE* was deleted) or even *fakA* alone (data not shown) in OG1RF. Growth of our *ΔfakB1,2,5* strain is also hindered in TSB, which has been used in *S. aureus* reports for examining effects of *fak* gene deletions and has been shown to contain a variety of fatty acids, including palmitic acid (C_16:0_), stearic acid (C_18:0_), and a monounsaturated C_18_ fatty acid, thus our phenotypes are not limited to BHI media (Fig. S5; [18, 20]). Furthermore, there is no transposon insertion in *fakA* within the OG1RF transposon library (22, 23). Another group reported they were not successful in the deletion of the entire *fakA* gene in *E. faecalis*, though they could generate a truncated *fakA* (24). The resulting morphology of this truncated *fakA* strain was remarkably similar to our *ΔfakB1,2,5* strain, supporting that defects in exogenous fatty acid utilization contribute to cellular phenotypes. It is possible that *E. faecalis* uses the Fak system for the appropriate placement of unsaturated vs saturated fatty acid tails onto either the *sn*-1 or *sn*-2 position of lipids. TesE was previously found to cleave oleic acid (unsaturated fatty acid) from ACP, which could result in its activation by the Fak system and its potential placement on *sn*-1 (13). We note that *E. faecalis* produces a significant amount of the unsaturated fatty acid *cis*-vaccenic acid, which is exactly the same length as oleic acid (Table 1 [1, 14, 15]). Perhaps TesE also cleaves *cis*-vaccenic acid from ACP, with the downstream consequence of altering its placement on a lipid headgroup. This may allow for optimal tail placement for membrane functionality, but clearly, more careful analyses of where fatty acids are placed and under what environmental conditions are needed.

The role of the Fak system in dictating membrane physical properties was unexpected. Previous work has noted that the addition of saturated fatty acids results in an overall decrease in the membrane fluidity of *E. faecalis* (15). However, the addition of palmitic acid to the *Δquint* strain had no impact on overall cellular fluidity. Our lipid analyses of the *Δquint* strain supplemented with ^13^C palmitic acid indicated that the fatty acid was free within the membrane but not on headgroups. This supports that membrane fluidity is driven by fatty acids placed on headgroups, and not free fatty acids within the membrane.

Remarkably little is known regarding how free fatty acids, vs localization of tail placement on headgroups, drive membrane biophysics. Extensive work in S*. aureus* with a *fakA* deletion strain, however, indicates altered signaling, metabolism, and virulence properties compared to a wild-type strain, supportive of key differences in cellular responses to free fatty acids within its membrane (9, 18, 25–30). Future analyses in *E. faecalis* are geared to better quantify the amount of fatty acid free within the membrane vs on headgroups, and the subsequent impacts on biophysical properties are underway to better dictate this behavior.

## MATERIALS AND METHODS

### Identification of FakA and FakB homologs

FakA and FakB homologs were identified using the NCBI Basic Local Alignment Search Tool for proteins (BLASTP). Briefly, each *S. aureus* (SAUSA300_0733 and SAUSA300_1318 [9]) and *S. pneumoniae* FakB protein (Sp1557, Sp1112, and Sp0742 [8]) was used as a query for BLASTP (protein-protein BLAST algorithm) against *E. faecalis* OG1RF using the non-redundant protein sequences (nr) database. Percent identity and percent similarity from each BLAST result were reported from the resulting alignments in Table S1.

### FakB protein modeling and multiple sequence alignment

The identified FakB homologs of *E. faecalis* (OG1RF_RS00080, OG1RF_RS07200, OG1RF_RS05020, and OG1RF_RS06660) along with saFakB1 were modeled using Protein Homology/Analogy Recognition Engine V 2.0 (Phyre^2^ [17]). Briefly, the amino acid sequence for each *E. faecalis* FakB homolog was entered into the server and modeled using intensive mode. A multiple sequence alignment of the FakB homologs of *E. faecalis* along with the FakB proteins of *S. aureus* and *S. pneumoniae* was aligned using Clustal Omega (31, 32). The resulting alignment was annotated by highlighting residues important for FakB function noted by Broussard et al. (16).

### Network analysis of DegV domain-containing proteins

Enzyme Function Initiative’s Enzyme Similarity Tool (EFI-EST, which draws networks of proteins and clusters them based on amino acid sequence similarity) was used to generate a representative node sequence similarity network (rep node SSN) for proteins within the DegV family (Pfam PF02645) (33). As this family is large (containing 34,797 proteins at the date of accession, 7 August 2021), we grouped sequences that shared ≥50% identity over 80% of the sequence into cluster IDs (UniRef50). Each of these cluster IDs is a node within the rep node SNN. Additionally, as we wished to gain insight into fatty acid binding specificities rather than additional functions, we trimmed the input sequences to the domain boundaries prior to generating the rep node SSN. Note that the annotated FakB proteins of *S. aureus* and *S. pneumoniae* only contain one domain, which makes up the majority (>95%) of the proteins, yet there are other instances where DegV is one domain within a multidomain protein. Fragments (sequences which do not contain a start and/or a stop codon) were not used in the analysis. An initial (permissive) BLAST E-value of 10^−5^ was used to calculate the similarity between cluster IDs. On calculating the similarities, the edges between cluster IDs in the rep node SSN were calculated using an alignment score of 55 (more stringent than the initial E-value; corresponding to ~35% identity between nodes). The EFI-EST Color SSN tool was used to assign cluster numbers.

### Network plot visualization

Networks were visualized and interpreted using Cytoscape v3.7.1 and Gephi V0.9.2 (34, 35). Nodes were arranged with the OpenOrd layout algorithm. As the FakB proteins of *S. aureus* and *S. pneumoniae*, along with the *E. faecalis* FakB homologues, were present in Cluster 1, we opted to analyze only this cluster. Cluster 1 originally contained 1,332 nodes, and 5,176 organisms were represented. Organisms that appeared within the network nodes were eliminated from further analysis if they represented >1 strain of that organism, or if they could not be identified as a single strain (for instance, if “marine sediment metagenome” or *Coprococcus* sp*.* were listed as an organism, they would be eliminated). Thus, the resulting network contained 3,423 organisms, 1,010 nodes, and 7,515 edges. To visualize subclusters within cluster 1, the modularity within the network was calculated in Gephi using a resolution of 1.25, which resulted in the differentiation of 52 subclusters within the network. The plot was colored using the resulting modularity classes.

Using the NCBI Taxonomy Browser and the Joint Genome Institution’s resources, each organism within the network was examined and manually curated for the type of organism (Gram stain, virus, or archaea), along with environmental origin, consisting of the following categories: animal gut, animal airways, animal oral cavity/throat, other animal sources, water sources, terrestrial sources, food sources, plant/algae sources, manmade object sources, multiple sources, atmospheric (air) sources, and other sources. Using Gephi V0.9.2, these categories were used to color the network plot (Fig. S4).

### Bacterial growth conditions

*Enterococcus faecalis* strains (Table S7) were grown statically in brain heart infusion medium (BHI; BD Difco) at 37°C. Overnight cultures were diluted to an optical density at 600 nm (OD_600_) of 0.01 prior to performing assays. OD_600_ nm was recorded over time as indicated. For the determination of generation times, fatty acids were supplemented at the following concentrations as indicated: oleic acid (C_18:1 *cis*9_) at 20 µg/mL (71 µM), linoleic acid (C_18:2 *cis*9,12_) at 10 µg/mL (35 µM), stearic acid (C_18:0_) at 20 µg/mL (40 µM), myristic acid (C_14:0_) at 5 µg/mL (22 µM), and palmitic acid (C_16:0_) at 5 µg/mL (19 µM) or 2.5 µg/mL (9.7 µM) as indicated in the text. For cerulenin outgrowth assays, cerulenin and oleic acid (C_18:1 *cis*9_) were supplemented at 5 µg/mL as previously described (14). For scanning electron microscopy and anisotropy experiments with fatty acid supplementation, cells were grown to mid-exponential phase (OD_600 nm_ ~0.25), and the indicated fatty acid was added to a final concentration of 5 µg/mL; this is referred to as “short-term exposure” (15, 36). Cells were then processed as indicated below for each assay. For all experiments utilizing fatty acids, a solvent control was also included, whereby an equal volume of ethanol was added to the control culture. All fatty acids and reagents were purchased from Millipore-Sigma unless otherwise noted.

For growth in laboratory-prepared chemically defined minimal-sm (CDM-sm) medium (see Table S4 for details; note a derivative of [37]), 2 mL of the overnight BHI culture was pelleted by centrifugation at 16,200 × *g* for 5 min and washed twice with 1× phosphate buffer saline (PBS) solution before resuspension in the same volume (2 mL) of CDM-sm medium. Cells were diluted to an OD_600_ of 0.01.

Counterselection was performed on M9 minimal medium containing yeast extract and glucose (M9MMYEG) agar plates (final concentrations 1× M9 salts, 0.25% yeast extract, 150 µg/mL 5-bromo-4-chloro-3-indolyl-β-D-galactopyranoside [X-gal], 0.5% glucose, and 10 mM *p-*Cl-phenylalanine [*p*-Cl-Phe]) (38). *Escherichia coli* cultures were grown in LB medium at 37°C with shaking. Antibiotics were used at the following concentrations when needed: erythromycin, 10 µg/mL (*E. faecalis*) or 100 µg/mL (*E. coli*); spectinomycin, 1,000 µg/mL; fusidic acid, 25 µg/mL; rifampicin, 250 µg/mL; chloramphenicol, 25 µg/mL. All media components, unless otherwise noted, were purchased from ThermoFisher.

### Generation of deletion strains

The strains and plasmids used in this study are listed in Table S7, and the sequences for oligonucleotides used are given in Table S8. Deletion strains were generated following the protocol of Kristich et al. (38). Briefly, to delete the *fakB* genes and *tesE*, ~1 kb upstream (designated as piece 1) and ~1 kb downstream (designated as piece 2) of the respective gene was amplified from *E. faecalis* OG1RF genomic DNA using primers with complementary overlaps with the vector (pCJK47) and the neighboring piece. The vector, pCJK47, was amplified with complementary overlaps to both piece 1 and piece 2. The amplified inserts and vectors were assembled using NEB Gibson Assembly Master Mix. The resulting assembly was transformed into *E. coli* EC1000. The resulting plasmids, pRDJ1, pRDJ2, pRDJ3, pRDJ4, and pRDJ5, used to generate *ΔfakB1, ΔfakB2, ΔfakB4, ΔfakB5,* and *ΔtesE,* respectively, were transformed into a conjugative donor strain of *E. faecalis*, CK111/pCF10-101. The resulting CK111/pCF10-101 derivatives were then mixed with an OG1RF recipient at a ratio of 1 part donor to 9 parts recipient. The mixture was plated on BHI, and mating was allowed to proceed for 5 h at 37°C. To select for transconjugant recipients, the resulting mixture was plated on BHI agar containing rifampicin, fusidic acid, erythromycin, and X-gal. Blue colonies from the recipient plates were then re-isolated on the same medium. The isolates were grown in BHI for 18–24 h, passaged again into fresh BHI, and plated on M9MMYEG. White colonies were isolated onto BHI, tested for erythromycin sensitivity, and screened for deletion of the respective gene.

### Ultra-high performance liquid chromatography, high-resolution mass spectrometry

Strains were grown as indicated to an OD_600_ of ~0.3, and 5 mL of cell culture was centrifuged (2,739 × *g* for 10 min). Cell pellets were resuspended and washed with 15 mL 1× PBS and stored at −80°C prior to lipid extraction. Lipids were extracted according to Woodall et al. (5).

Lipidome analysis was performed as previously described (3–5). In brief, an UltiMate 3000 ultra-high performance liquid chromatography system (UHPLC, Dionex, Sunnyvale, CA) was used to inject 10 µL of sample onto a CORTECS C18 column (90 Å, 2.7 µm, 2.1 mm × 150 mm; Waters) controlled at 40°C. Mobile phase A was 60:40 acetonitrile:water with 10 mM ammonium formate as a buffer and 0.1% formic acid, while mobile phase B consisted of 90:10 2-propanol:acetonitrile with 10 mM ammonium formate as a buffer and 0.1% formic acid. Eluent was introduced to the mass spectrometer via an electrospray ionization source, and mass analysis was performed using an Exactive Plus (Thermo Scientific, Waltham, MA) mass spectrometer operated in dual polarity mode. Ions of respective DAG and MGDG species were detected in positive mode, while free fatty acids, phosphatidylglycerol, lysyl-phosphatidylglycerol, and cardiolipin were detected in negative mode. Masses were detected in full scan mode within a scan range of 100–1,500 *m/z*, operated at a resolution of 140,000, with an automatic gain control target of 3 × 10^6^ ions, and a maximum IT time of 100 ms. Full scan data were complemented with all ion fragmentation data at a resolution of 35,000 utilizing 35 eV collisional energy. The mass spectrometer was calibrated every 24 h.

Statistical analysis and data processing also followed the same procedure as prior (3–5). Raw files from Xcalibur were converted to .mzML format via MS convert (ProteoWizard) and then imported into El MAVEN, where the area under the curve for mass to charge (*m/z*) values matching the retention time of an in-house curated lipid library were integrated with a mass error of less than 5 ppm. Ion intensities from integration and retention time data were analyzed in Excel, and data visualizations were performed in R Studio.

### Gas chromatography-fatty acid methyl ester analysis

GC-FAME analysis was performed as previously described (1). Briefly, *E. faecalis* strains were grown as indicated in the text until mid-log phase (OD_600_ ~0.3–0.4), and cellular aliquots (10 mL) were harvested. Cell pellets were washed with 1× PBS twice to remove the media. The cell pellets were stored at −80°C prior to GC-FAME analysis, which was performed by Microbial ID, Inc. (Newark, DE).

### Synthesis of ^13^C palmitic acid

The synthesis of palmitic acid (U-^13^C) used in this study was performed with modifications to previously published methods (39). To a solution of methyl palmitate (U-^13^C) (0.88 g, 3.07 mmol) in 45 mL tetrahydrofuran/ethanol/water (THF/EtOH/H_2_O; 1:1:1) was added sodium hydroxide (NaOH, 0.37 g, 9.3 mmol) and allowed to stir for 30 h at 70°C. Once no change was observed on thin layer chromatography, ethyl acetate (EtOAc, 45 mL) was added, and the solution was washed with 1 M hydrochloric acid (HCl, 45 mL), water (45 mL), and brine (45 mL). The aqueous layers were extracted with EtOAc (20 mL), and the organic layers were combined, dried with magnesium sulfate (MgSO_4_), filtered, and the filtrate was concentrated to give a white solid. Spectral data can be found in Fig. S6 and S7.

### Scanning electron microscopy

Strains were grown as described above, with short-term fatty acid supplementation for 30 min as indicated. For growth in CDM, SM cells were grown overnight in BHI and washed three times in 1× PBS before resuspension in CDM. Cells were harvested at an OD600 of ~0.3 and centrifuged at 3,019 × *g* for 10 min and washed twice with an equivalent volume of 1× PBS. Pelleted cells were then resuspended in 500 µL of the primary fixative (3% glutaraldehyde in 0.1 M cacodylate 1× PBS), vortexed, and incubated for 1 h at room temperature. Samples were centrifuged for 10 min at 15,115 × *g*, rinsed with 1× PBS three times consecutively, and then resuspended in 200 µL of 2% osmium tetroxide in 0.1 M cacodylate (1× PBS) and incubated for 1 h room temperature. Samples were then pelleted and washed in deionized water three times as above. The fixed cells were resuspended in 1× PBS and kept at 4°C overnight.

Silicone square chips were washed with methanol and coated with 20 µL of 0.1 mg/mL Poly-L-Lysine for 2 min, followed by removal with compressed air. The overnight samples were centrifuged at 15,115 × *g* for 5 min. A total of 20 µL of cells was applied to the surface of the silicone chips and incubated at room temperature for 25 min. The silicone chips were then dehydrated by a series of ethanol steps (25%, 50%, 75%, 95%, and 100%) for 10 min each. After the dehydration series, the cells on the silicone chips were put into a Critical Point Dryer. Chips were then sputter-coated with iridium (Ir) for 20 s at 20 mA. A Zeiss Auriga FIB-SEM microscope at the University of Tennessee Institute for Advanced Materials and Manufacturing (IAMM) was used to capture images. A minimum of 10 images for each biological replicate were taken. For morphological analysis, a minimum of 100 cells per replicate were categorized as either diplococci or non-diplococci. Cells were only counted if the entire cell could be seen in the image.

### Anisotropy

Anisotropy was performed as described with the following modifications (15). A total of 5 mL cells from short-term exposure to 5 µg/mL of myristic or palmitic acids and respective solvent control (see above) were treated with a final concentration of 7 µM 1,6-diphenyl-1,3,5-hexatriene for 30 min at 37°C. Cells were washed twice with 10 mL 1× PBS at 3,019 × *g* for 10 min. Cells were resuspended in a final volume of 10 mL 1× PBS, and the *r* value (fluidity) was measured via an Agilent Technologies Cary Eclipse Fluorescent Spectrophotometer at an excitation wavelength of 350 nm emission wavelength of 428 nm. The observed biological range of values for anisotropy using DPH is 0.1 to 0.3, with a lower number equivalent to a more fluid membrane.

### Statistical analyses

For statistical analyses, Welch’s *t*-test was employed via GraphPad Prism version 10.03 for Macintosh, GraphPad Software, Boston, Massachusetts, USA, https://www.graphpad.com/.

## SUPPLEMENTAL MATERIAL

Supplemental tables and figures jb.00121-25-s0001.docx Tables S1 to S8 and Fig. S1 to S7.

ASM does not own the copyrights to Supplemental Material that may be linked to, or accessed through, an article. The authors have granted ASM a non-exclusive, world-wide license to publish the Supplemental Material files. Please contact the corresponding author directly for reuse.
