# Supplementary material for: Unexpected contribution of the Fak system and the thioesterase TesE to the growth and membrane physiology of Enterococcus faecalis
Source: J Bacteriol. 2025 Jun 30;207(7):e00121-25. doi: 10.1128/jb.00121-25 (PMC12288450; doi:10.1128/jb.00121-25)
Supplement: Supplemental tables and figures — Tables S1 to S8 and Fig. S1 to S7. [file jb.00121-25-s0001.docx]

**Supplemental Materials and Methods**

**Synthesis of U-^13^C palmitic acid (C_16:0_)**

**General Methods:** All reactions were performed under inert atmosphere. All solvents and chemicals were purchased through either Fisher Scientific, VWR, or Cambridge Isotope Laboratories and were reagent grade. Thin-layer chromatography (TLC) was carried out using Sorbent Technologies silica G w/UV254 TLC plates. Compounds were visualized as single spots using permanent staining of TLC spots achieved using Basic KMnO_4_. NMRs were taken on Varian Inova 500 MHz instrument (Palo Alto, CA) in solutions of CDCl_3_. High resolution mass spectrum were performed on Exactive Plus mass spectrometer using electrospray ionization.

**Preparation of Palmitic Acid (U-^13^C).** To a solution of methyl palmitate (U-^13^C) (0.88 g, 3.07 mmol) in 45 mL THF/EtOH/H_2_O (1:1:1) was added NaOH (0.37 g, 9.3 mmol) and allowed to stir for 30 h at 70^o^C. Once no change was observed on TLC, EtOAc (45 mL) was added and the solution was washed with 1M HCl (45 mL), water (45 mL), and brine (45 mL). The aqueous layers were extracted with EtOAc (20 mL) and the organic layers were combined, dried with MgSO_4_, filtered, and the filtrate was concentrated to give a white solid.

**Methyl Palmitate (U-^13^C):** white solid obtained in 93.4% yield. TLC (Silica G w/ Basic KMnO_4_) R_f_ = 0.74, 10:90 EtOAc:Hex;  ^1^H NMR (500 MHz, CDCl_3_) (exchangeable proton not observed) δ 2.34 (dd, *J* = 129.5, 5.6 Hz, 2H), 1.62 (d, *J* = 139.6 Hz, 2H), 1.25 (d, *J* = 118.4 Hz, 24H), 1.04 – 0.66 (m, 3H). ^13^C NMR (126 MHz, CDCl_3_) δ 180.13, 179.69, 34.36, 34.09, 33.92, 33.65, 32.19, 31.92, 31.69, 29.65, 29.26, 29.00, 24.93, 24.66, 24.38, 22.95, 22.68, 22.40, 14.22, 13.95. HRMS (ESI) m/z: [M-H]^-^ Calcd for ^13^CH_3_(^13^CH_2_)_14_^13^CO_2_H, 271.2861; found, 271.2867.

**Table S1. Percent identity and positive substitutions of *Enterococcus faecalis* FakB proteins to *Staphylococcus aureus* and *Streptococcus pneumoniae* FakB proteins as determined by NCBI BLAST.**

| ***E. faecalis* FakB loci and proteins** | **Percent identity** | **Percent positive substitutions** |
| --- | --- | --- |
| *S. aureus* FakB1 (saturated specificity) | | |
| OG1RF_ RS06660 (FakB1) | 40% | 60% |
| OG1RF_ RS05020 (FakB5) | 28% | 51% |
| OG1RF_ RS07200 (FakB2) | 26% | 51% |
| OG1RF_ RS00080 (FakB4) | 26% | 50% |
| *S. aureus* FakB2 (unsaturated specificity) | | |
| OG1RF_ RS07200 (FakB2) | 37% | 58% |
| OG1RF_ RS06660 (FakB1) | 33% | 55% |
| OG1RF_ RS00080 (FakB4) | 31% | 53% |
| OG1RF_ RS05020 (FakB5) | 29% | 51% |
| *S. pneumoniae* FakB1 (saturated specificity) | | |
| OG1RF_ RS06660 (FakB1) | 41% | 61% |
| OG1RF_ RS05020 (FakB5) | 27% | 49% |
| OG1RF_ RS00080 (FakB4) | 27% | 48% |
| *S. pneumoniae* FakB2 (monounsaturated specificity) | | |
| OG1RF_ RS07200 (FakB2) | 54% | 73% |
| OG1RF_ RS06660 (FakB1) | 27% | 50% |
| OG1RF_ RS00080 (FakB4) | 26% | 49% |
| *S. pneumoniae* FakB3 (polyunsaturated specificity) | | |
| OG1RF_RS07200 (FakB2) | 20% | 46% |

**Table S2. Generation times of *E. faecalis* strains grown in BHI (rich media).**

P-values for each deletion strain compared to wild-type OG1RF calculated using Welch’s T-test. *n* = 3 replicates.

| **Media Supplement and Concentration^a^** | **Strain** | **Generation Time (Minutes)** | ***P*-values against OG1RF** |
| --- | --- | --- | --- |
| No supplement | OG1RF | 33.6 ± 4.8 | NA |
|  | *ΔtesE* | 31.8 ± 1.6 | NA |
|  | *ΔfakB1,2,5* | 56.8 ± 6.9 | 0.03 |
|  | *ΔfakB1,2,5*/*ΔtesE* | 50.9 ± 6.6 | 0.02 |
|  | *Δquint* | 31.9 ± 3.3 | 0.09 |
| Ethanol^a^ | OG1RF | 37.6 ± 0.5 | NA |
|  | *ΔfakB1* | 39.1 ± 0.8 | 0.10 |
|  | *ΔfakB2* | 40.7 ± 0.7 | 0.01 |
|  | *ΔfakB4* | 38.6 ± 1.2 | 0.40 |
|  | *ΔfakB5* | 39.9 ± 0.4 | P < 0.01 |
|  | *ΔfakB1,2* | 42.4 ± 0.0 | P < 0.01 |
|  | *ΔfakB1,5* | 37.3 ± 2.8 | 0.88 |
|  | *ΔfakB2,4* | 38.1 ± 0.8 | 0.49 |
|  | *ΔfakB4,5* | 31.2 ± 5.4 | 0.23 |
|  | *ΔfakB1,2,4* | 45.5 ± 0.8 | P < 0.01 |
|  | *ΔfakB1,2,5* | 46.2 ± 1.4 | P < 0.01 |
|  | *ΔfakB1,4,5* | 36.9 ± 2.4 | 0.72 |
|  | *ΔtesE* | 38.5 ± 0.3 | 0.12 |
|  | *ΔfakB1,2,5*/*ΔtesE* | 46.5 ± 0.7 | P < 0.01 |
|  | *Δquint* | 39.5 ± 1.0 | 0.09 |
| C_18:2_ *_cis_*_9,12_^b^  (linoleic acid) | OG1RF | 66.0 ± 15.2 | NA |
|  | *ΔfakB1* | 57.4 ± 5.4 | 0.27 |
|  | *ΔfakB2* | 49.7 ± 11.1 | 0.06 |
|  | *ΔfakB4* | 58.3 ± 13.3 | 0.35 |
|  | *ΔfakB1,2* | 86.4 ± 5.7 | 0.05 |
|  | *ΔfakB2,4* | 51.9 ± 5.4 | 0.09 |
|  | *ΔfakB1,2,4* | 86.0 ± 5.8 | 0.04 |
|  | *ΔfakB1,2,5* | 135.3 ± 9.6 | P < 0.01 |
|  | *ΔtesE* | 81.4 ± 8.9 | 0.15 |
|  | *ΔfakB1,2,5*/*ΔtesE* | 118.6 ± 2.4 | P < 0.01 |
|  | *Δquint* | 75.7 ± 1.4 | 0.22 |
| C_18:1_ *cis*_9_^c^  (oleic acid) | OG1RF | 37.9 ± 0.6 | NA |
|  | *ΔfakB1* | 35.5 ± 1.5 | 0.14 |
|  | *ΔfakB2* | 35.3 ± 0.7 | 0.01 |
|  | *ΔfakB4* | 36.4 ± 1.7 | 0.35 |
|  | *ΔfakB1,2* | 58.2 ± 3.0 | 0.008 |
|  | *ΔfakB2,4* | 35.9 ± 0.9 | 0.07 |
|  | *ΔfakB1,2,4* | 67.1 ± 4.9 | 0.01 |
|  | *ΔfakB1,2,5* | 92.6 ± 4.9 | P < 0.01 |
|  | *ΔtesE* | 37.5 ± 1.1 | 0.71 |
|  | *ΔfakB1,2,5*/*ΔtesE* | 85.8 ± 2.7 | P < 0.01 |
|  | *Δquint* | 37.9 ± 8.6 | 1.0 |
| C_18:0_^d^  (Stearic acid) | OG1RF | 82.0 ± 12.5 | NA |
|  | *ΔfakB1* | 107.3 ± 16.0 | 0.137 |
|  | *ΔfakB4* | 84.8 ± 5.1 | 0.68 |
|  | *ΔfakB5* | 107.0 ± 8.3 | 0.02 |
|  | *ΔfakB1,5* | 162.0 ± 10.8 | P < 0.01 |
|  | *ΔfakB4,5* | 94.4 ± 13.7 | 0.34 |
|  | *ΔfakB1,2,5* | 60.3 ± 6.8 | 0.02 |
|  | *ΔfakB1,4,5* | 185.9 ± 28.1 | 0.03 |
|  | *ΔtesE* | 63.0 ± 7.2 | 0.04 |
|  | *ΔfakB1,2,5*/*ΔtesE* | 62.3 ± 11.2 | 0.11 |
|  | *Δquint* | 37.9 ± 1.4 | P < 0.01 |
| C_16:0_^e^  (Palmitic acid) | *Δquint* | 43.8 ± 0.9 | NA |
| C_14:0_ (Myristic acid)^f^ | *Δquint* | 44.6 ± 1.3 | NA |
| C_16:0_  (Palmitic acid)^g^ | *ΔfakB1,2,5* | 120.2 ± 10.6 | NA |
|  | *ΔfakB1,2,5*/*ΔtesE* | 115.7 ± 16.1 | NA |
|  | *Δquint* | 40.8 ± 1.7 | NA |

^a^Ethanol: 0.1% final (solvent control for fatty acid supplements)

^b^Linoleic acid: 10 µg/mL

^c^Oleic acid: 20 µg/mL

^d^Stearic acid: 20 µg/mL

^e^Palmitic acid: 5 µg/mL; at this concentration, wild-type OG1RF reaches an early growth stasis followed by the outgrow of a suppressor as previously reported (4, 5).

^f^Myristic acid: 5 µg/mL; at this concentration, wild-type OG1RF reaches an early growth stasis followed by the outgrow of a suppressor as previously reported (4, 5).

^g^Palmitic acid: 2.5 µg/mL

**Table S3. Generation times of *E. faecalis* strains grown in CDM-Sm.**

P-values for each deletion strain compared to wild-type OG1RF calculated using Welch’s T-test. White cells indicate no difference. *n* = 3 replicates.

| **Supplement and Concentration^a^** | **Strain** | **Generation Time (Minutes)** | ***P*-values against OG1RF** |
| --- | --- | --- | --- |
| Ethanol^a^ | OG1RF | 55.8 ± 3.9 | NA |
|  | *ΔfakB1,2,5* | 57.7 ± 1.0 | 0.48 |
|  | *ΔfakB1,2,5*/*ΔtesE* | 55.4 ± 2.5 | 0.89 |
|  | *Δquint* | 50.1 ± 9.2 | 0.41 |
| C_18:1_ *cis*_9_^b^  (oleic acid) | OG1RF | 108.0 ± 25.1 | NA |
|  | *ΔfakB1,2,5* | 245.2 ± 29.0 | 0.033 |
|  | *ΔfakB1,2,5*/*ΔtesE* | 219.5 ± 95.7 | 0.20 |
|  | *Δquint* | 65.1 ± 23.1 | 0.063 |

^a^Ethanol: 0.1% final (solvent control for fatty acid supplements)

^b^Oleic acid: 20 µg/mL

**Table S4. Chemically defined minimal-sm (CDM-sm) medium for *E. faecalis*^a^**

| **Component** | **Media concentration** | **Component** | **Media concentration** |
| --- | --- | --- | --- |
| K_2_HPO_4_ | 7g/L | Lysine HCl | 20 mg/L |
| KH_2_PO_4_ | 2g/L | Methionine | 20 mg/L |
| Sodium citrate | 0.5g/L | Proline | 20 mg/L |
| MgSO_4_ | 0.1g/L | Serine | 20 mg/L |
| (NH_4_)_2_SO_4_ | 1g/L | Leucine | 20 mg/L |
| Thiamine | 100 mg/L | Phenylalanine | 20 mg/L |
| Adenine | 20 mg/L | Threonine | 20 mg/L |
| Guanine | 20 mg/L | Glycine | 20 mg/L |
| Thymine | 20 mg/L | Alanine | 20 mg/L |
| Uracil | 20 mg/L | Asparagine | 20 mg/L |
| Biotin | 20 mg/L | Cysteine | 20 mg/L |
| Calcium chloride | 46 μM | Aspartic acid | 20 mg/L |
| Pantothenic acid | 20 mg/L | Glutamic acid | 20 mg/L |
| Pyridoxine | 20 mg/L | Isoleucine | 20 mg/L |
| Nicotinic acid | 2 mg/L | Tryptophan | 20 mg/L |
| Riboflavin | 2 mg/L | Tyrosine | 20 mg/L |
| Folic acid | 200 μg/L | Valine | 20 mg/L |
| Glucose | 0.20% | FeSO_4_ | 5.68 g/L |
| Arginine | 20 mg/L | Sodium Bicarbonate | 2.5 g/L |
| Glutamine | 20 mg/L |  |  |
| Histidine HCl | 20 mg/L |  |  |
| Lysine HCl | 20 mg/L |  |  |
| Methionine | 20 mg/L |  |  |
| Proline | 20 mg/L |  |  |
| Serine | 20 mg/L |  |  |
| Leucine | 20 mg/L |  |  |
| Phenylalanine | 20 mg/L |  |  |

^a.^ Modified from (3).

**Table S5. Detection of ^13^C oleic acid upon growth supplementation of OG1RF derived strains.**

| **Percent ^13^C incorporation^a^** | | | |
| --- | --- | --- | --- |
| **Lipid species** | **OG1RF** | ***ΔfakB1,2,5*** | ***Δquint*** |
| C_18:1_ | 97.95% | 97.46% | 96.67% |
| C_19:1/cyclo_ | 95.63% | 92.01% | 90.37% |
| C_20:1_ | 49.52% | 14.06% | 0.00% |
| CL 62:1 | 29.81% | 7.30% | 0.00% |
| CL 64:1 | 25.89% | 7.62% | 0.00% |
| CL 64:2 | 54.70% | 19.28% | 0.00% |
| CL 66:0 | 34.65% | 7.84% | 0.00% |
| CL 66:1 | 17.43% | 5.81% | 0.00% |
| CL 66:2 | 49.51% | 16.92% | 0.00% |
| CL 68:1 | 32.21% | 9.51% | 0.00% |
| CL 68:2 | 32.48% | 9.83% | 0.00% |
| CL 68:3 | 76.46% | 42.64% | 0.00% |
| CL 68:5 | 63.36% | 22.25% | 0.00% |
| CL 70:2 | 62.48% | 19.27% | 0.00% |
| CL 70:3 | 80.43% | 48.45% | 0.00% |
| CL 70:5 | 31.47% | 8.79% | 0.00% |
| CL 72:4 | 98.67% | 94.06% | 0.00% |
| CL 72:6 | 76.91% | 38.70% | 0.00% |
| PG 30:1 | 58.53% | 18.76% | 0.00% |
| PG 34:1 | 57.56% | 24.50% | 0.00% |
| PG 34:2 | 85.15% | 39.52% | 0.00% |
| PG 35:2 | 84.94% | 28.62% | 0.00% |
| PG 36:1 | 61.12% | 19.74% | 0.00% |
| PG 36:2 | 96.15% | 86.66% | 0.00% |
| PG 37:2 | 86.30% | 36.18% | 0.00% |

^a^ Percent ^13^C incorporation was determined via the ion count of ^13^C labeled mass/ ion count of all masses summed (including ^12^C). Cells were exposed to ^13^C oleic acid for 30 mins in exponential phase.

**Table S6. Detection of ^13^C palmitic acid upon growth supplementation of OG1RF derived strains.**

| Percent ^13^C palmitic acid incorporation^a^ | | | | | | |
| --- | --- | --- | --- | --- | --- | --- |
|  | Short term (5μg/mL)^b^ | | | Long term (2.5μg/mL)^c^ | | |
| Lipid species | OG1RF | *ΔfakB1,2,5* | *Δquint* | OG1RF | *ΔfakB1,2,5* | *Δquint* |
| C16:0 | 6% | 8% | 9% | 4% | 11% | 10% |
| CL 60:0 | 54% | 36% | 0% | 96% | 69% | 0% |
| CL 60:1 | 42% | 16% | 0% | 94% | 38% | 00% |
| CL 62:0 | 73% | 48% | 3% | 98% | 64% | 1% |
| CL 62:1 | 52% | 29% | 0% | 95% | 47% | 0% |
| CL 62:2 | 36% | 19% | 0% | 92% | 31% | 0% |
| CL 64:0 | 91% | 61% | 0% | 100% | 71% | 0% |
| CL 64:1 | 67% | 36% | 0% | 97% | 25% | 0% |
| CL 64:2 | 45% | 26% | 0% | 92% | 35% | 0% |
| CL 66:0 | 80% | 52% | 0% | 93% | 54% | 7% |
| CL 66:1 | 83% | 37% | 0% | 98% | 59% | 2% |
| CL 66:2 | 52% | 25% | 0% | 91% | 39% | 0% |
| CL 66:3 | 31% | 14% | 0% | 72% | 22% | 0% |
| CL 68:0 | 63% | 70% | 0% | 57% | 31% | 0% |
| CL 68:1 | 64% | 37% | 0% | 82% | 44% | 0% |
| CL 68:2 | 58% | 27% | 0% | 89% | 45% | 0% |
| CL 68:3 | 37% | 18% | 0% | 70% | 23% | 0% |
| LPG 32:0 | 92% | 53% | 0% | 94% | 39% | 6% |
| LPG 32:1 | 43% | 17% | 0% | 69% | 16% | 0% |
| LPG 34:0 | 69% | 40% | 0% | 72% | 25% | 1% |
| LPG 34:1 | 56% | 25% | 0% | 70% | 22% | 0% |
| PG 28:0 | 28% | 21% | 0% | 73% | 25% | 5% |
| PG 30:0 | 71% | 30% | 0% | 77% | 26% | 0% |
| PG 30:1 | 39% | 10% | 0% | 74% | 8% | 0% |
| PG 32:0 | 95% | 56% | 0% | 93% | 39% | 0% |
| PG 32:1 | 59% | 25% | 0% | 73% | 21% | 0% |
| PG 33:0 | 71% | 30% | 0% | 77% | 26% | 0% |
| PG 34:0 | 74% | 38% | 0% | 76% | 26% | 0% |
| PG 34:1 | 59% | 26% | 0% | 71% | 22% | 0% |
| PG 35:1 | 36% | 14% | 0% | 65% | 25% | 0% |

^a^ Percent ^13^C incorporation was determined via the ion count of ^13^C labeled mass/ ion count of all masses summed (including ^12^C).

^b^ Cells were exposed to ^13^C palmitic acid for 30 mins in exponential phase.

^c^ Cells were exposed to ^13^C palmitic acid from lag phase through exponential phase.

**Table S7. Bacterial strains and plasmids used in this study.**

| **Strain** | **Relevant genotype or description** | **Source** |
| --- | --- | --- |
| *Escherichia coli* EC1000 | Cloning strain for *repA*-dependent plasmids | G. Dunny, University of Minnesota |
| *E. faecalis* CK111/pCJ10-101 | Conjugative donor strain; harbors non-transferable pCJ10 derivative plasmid  Spec^R^ | Kristich et al. (2) |
| *Enterococcus faecalis* OG1RF | Laboratory strain  Rif^R^, Fus^R^ | Lab stock |
|  | Derivative of OG1RF deleted for *fakB1*; OG1RF_11289 or OG1RF_RS06660 | This study |
| *ΔfakB2* | Derivative of OG1RF deleted for *fakB2*; OG1RF_11397 or OG1RF_RS07200 | This study |
| *ΔfakB4* | Derivative of OG1RF deleted for *fakB4*;  OG1RF_10014 or OG1RF_RS00080 | This study |
| *ΔfakB5* | Derivative of OG1RF deleted for *fakB5*;  OG1RF_10963 or OG1RF_RS05020 | This study |
| *ΔtesE* | Derivative of OG1RF deleted for *tesE*  OG1RF_10252O; G1RF_RS01405; | This study |
| *ΔfakB1,2* | Derivative of *ΔfakB2* | This study |
| *ΔfakB1,5* | Derivative of *ΔfakB5* | This study |
| *ΔfakB1,2,4* | Derivative of *ΔfakB1,2* | This study |
| *ΔfakB1,4.5* | Derivative of *ΔfakB1,5* | This study |
| *ΔfakB4,5* | Derivative of *ΔfakB5* | This study |
| *ΔfakB2,4* | Derivative of *ΔfakB4* | This study |
| *ΔfakB1,4* | Derivative of *ΔfakB4* | This study |
| *ΔfakB1,2,5* | Derivative of *ΔfakB1,5* | This study |
| *ΔfakB2,5* | Derivative of *ΔfakB5* | This study |
| *ΔfakB2,4,5* | Derivative of *ΔfakB4,5* | This study |
| *ΔfakB1,4,5/ΔtesE* | Derivative of *ΔfakB1,4,5* | This study |
| *ΔfakB2,5* | Derivative of *ΔfakB5* | This study |
| *ΔfakB2,4,5* | Derivative of *ΔfakB2,4* | This study |
| *ΔfakB1,2/ΔtesE* | Derivative of *ΔfakB1,2* | This study |
| *ΔfakB1,2,5/ΔtesE* | Derivative of*ΔfakB1,2,5* | This study |
| *Δquint (ΔfakB1,2,4,5/ΔtesE)* | Derivative of *ΔfakB1,2,5/ΔtesE* | This study |
|  |  |  |
| *Escherichia coli* EC1000 | Cloning strain for *repA*-dependent plasmids | G. Dunny, University of Minnesota |
| *E. faecalis* CK111/pCJ10-101 | Conjugative donor strain; harbors non-transferable pCJ10 derivative plasmid  Spec^R^ | Kristich et al. (2) |
| **Plasmid** | | |
| pCJK47 | Used for markerless exchange | Kristich et al. (2) |
| pRDJ1 | pCJK47 derivative containing flanking regions of OG1RF_RS06660 (*fakB1*) | This work |
| pRDJ2 | pCJK47 derivative containing flanking regions of OG1RF_RS07200 (*fakB2*) | This work |
| pRDJ3 | pCJK47 derivative containing flanking regions of OG1RF_RS00080 (*fakB4*) | This work |
| pRDJ4 | pCJK47 derivative containing flanking regions of OG1RF_RS05020 (*fakB5*) | This work |
| pRDJ5 | pCJK47 derivative containing flanking regions of OG1RF_RS01405 (*tesE*) | This work |

**Table S8. Oligonucleotides used in this study.**

| **Name** | **Sequence** | **Use** |
| --- | --- | --- |
| EF1492 | GTGATACACATGGAAGCAATTCGTCGTGTTGCCCGGGTACCATGGCATGCTAAGCTTGATTTTCGTTC | Generate overhangs on pCJK47 for *fakB2* |
| EF1493 | GAAAGAATGGATAACCAAATCAGAAGGTAGTCTAGAACTAGCGATTCTGAAATCACCATTTAAAAAACTC | Generate overhangs on pCJK47 for *fakB2* |
| EF1494 | GAGTTTTTTAAATGGTGATTTCAGAATCGCTAGTTCTAGACTACCTTCTGATTTGGTTATCCATTCTTTC | Generate overhangs on *fakB2* piece 1 insert |
| EF1495 | CTTTTAAAGAAAGAGTGATAAATTTATGACAAACGCTATACAGACTAAGAAATAACGAGGTTGGGTCTCAC | Generate overhangs on *fakB2* piece 1 insert |
| EF1496 | GTGAGACCCAACCTCGTTATTTCTTAGTCTGTATAGCGTTTGTCATAAATTTATCACTCTTTCTTTAAAAG | Generate overhangs on *fakB2* piece 2 inserts |
| EF1497 | GAACGAAAATCAAGCTTAGCATGCCATGGTACCCGGGCAACACGACGAATTGCTTCCATGTGTATCAC | Generate overhangs on *fakB2* piece 2 insert |
| EF1564 | CTGAGTCAGCGATGAATGCCACTTTATCCCCGGGTACCATGGCATGCTAAGCTTGATTTTCGTTC | Generate overhangs on pCJK47 for *fakB4* |
| EF1512 | GATTCCCACCCGTGCTGCTTTATCCATGTCTAGAACTAGCGATTCTGAAATCACCATTTAAAAAACTC | Generate overhangs on pCJK47 for *fakB4* |
| EF1511 | GAGTTTTTTAAATGGTGATTTCAGAATCGCTAGTTCTAGACATGGATAAAGCAGCACGGGTGGGAATC | Generate overhangs on *fakB4* piece 1 insert |
| EF1565 | CAAAAGAAAACACAGCAATACAACCTATTCCGGGACAATTGCCCCCTTCATGTTTTCTTTTATTTTAC | Generate overhangs on *fakB4* piece 1 insert |
| EF1566 | GTAAAATAAAAGAAAACATGAAGGGGGCAATTGTCCCGGAATAGGTTGTATTGCTGTGTTTTCTTTTG | Generate overhangs on *fakB4* piece 2 insert |
| EF1567 | GAACGAAAATCAAGCTTAGCATGCCATGGTACCCGGGGATAAAGTGGCATTCATCGCTGACTCAG | Generate overhangs on *fakB4* piece 2 insert |
| EF1526 | GAAAGTATCAAATAAGATTAGAAGGAAAAAGAGCCCGGGTACCATGGCATGCTAAGCTTGATTTTCGTTC | Generate overhangs on pCJK47 for *fakB5* |
| EF1527 | CATCAACGTAACTAGTAATACGCCATAGCACTCTAGAACTAGCGATTCTGAAATCACCATTTAAAAAACTC | Generate overhangs on pCJK47 for *fakB5* |
| EF1528 | GAGTTTTTTAAATGGTGATTTCAGAATCGCTAGTTCTAGAGTGCTATGGCGTATTACTAGTTACGTTGATG | Generate overhangs on *fakB5* piece 1 insert |
| EF1529 | CAAAAAAATTTGAGGGGATTTTTCATTTAATTAATCGTGCGATCTTTTCTTTATTCATGTGTCATCTCATC | Generate overhangs on *fakB5* piece 1 insert |
| EF1530 | GATGAGATGACACATGAATAAAGAAAAGATCGCACGATTAATTAAATGAAAAATCCCCTCAAATTTTTTTG | Generate overhangs on *fakB5* piece 2 insert |
| EF1531 | GAACGAAAATCAAGCTTAGCATGCCATGGTACCCGGGCTCTTTTTCCTTCTAATCTTATTTGATACTTTC | Generate overhangs on *fakB5* piece 2 insert |
| EF1536 | GAAAAACGACAACACTTTGCAGAACGTATGCCCGGGTACCATGGCATGCTAAGCTTGATTTTCGTTC | Generate overhangs on pCJK47 for *fakB1* |
| EF1537 | GAACCGTTGCCACCACCTGAATCTGTTTGTCTAGAACTAGCGATTCTGAAATCACCATTTAAAAAACTC | Generate overhangs on pCJK47 for *fakB1* |
| EF1538 | GAGTTTTTTAAATGGTGATTTCAGAATCGCTAGTTCTAGACAAACAGATTCAGGTGGTGGCAACGGTTC | Generate overhangs on *fakB1* piece 1 insert |
| EF1539 | GAGACAGAAGTAGTTTACTTTTGTCTCATCCATTTTGTAACCTCGTTTCACTAATTTTC | Generate overhangs on *fakB1* piece 1 insert |
| EF1540 | GAAAATTAGTGAAACGAGGTTACAAAATGGATGAGACAAAAGTAAACTACTTCTGTCTC | Generate overhangs on *fakB1* piece 2 insert |
| EF1541 | GAACGAAAATCAAGCTTAGCATGCCATGGTACCCGGGCATACGTTCTGCAAAGTGTTGTCGTTTTTC | Generate overhangs on *fakB1* piece 2 insert |
| EF1877 | CTTGAATTTGAGTTACGATGATTTGAATCCCCCGGGTACCATGGCATGCTAAGCTTGATTTTCGTTC | Generate overhangs on pCJK47 for *tesE* |
| EF1878 | CTTTCAGAAGTAAATCAAGCCTACAATGTCTCTAGAACTAGCGATTCTGAAATCACCATTTAAAAAACTC | Generate overhangs on pCJK47 for *tesE* |
| EF1873 | GAGTTTTTTAAATGGTGATTTCAGAATCGCTAGTTCTAGAGACATTGTAGGCTTGATTTACTTCTGAAAG | Generate overhangs on *tesE* piece 1 insert |
| EF1874 | GACTTTCGACTTCTTGGCCATATTCTACCCCACGAAATAATCTCCTTTTTCCAATG | Generate overhangs on *tesE* piece 1 insert |
| EF1875 | CATTGGAAAAAGGAGATTATTTCGTGGGGTAGAATATGGCCAAGAAGTCGAAAGTC | Generate overhangs on *tesE* piece 2 insert |
| EF1876 | GAACGAAAATCAAGCTTAGCATGCCATGGTACCCGGGGGATTCAAATCATCGTAACTCAAATTCAAG | Generate overhangs on *tesE* piece 2 insert |


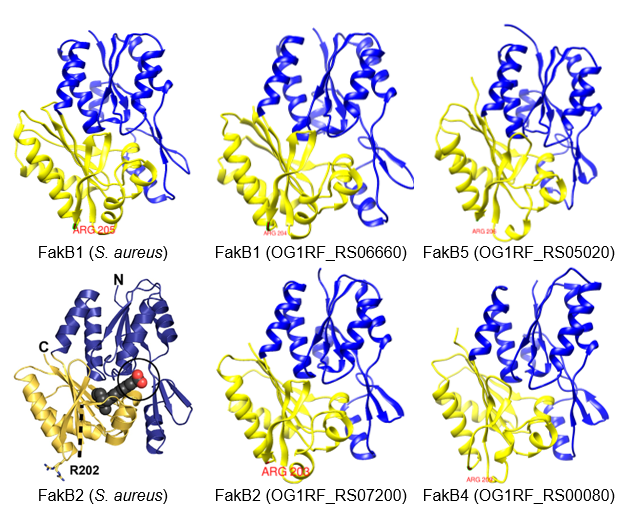


**Fig S1. Predicted structural models of FakB homologs generated by Phyre^2^.**

Phyre^2^ models were generated for the FakB homologs of *E. faecalis* and FakB1 of *S. aureus.* The models are depicted alongside the published crystal structure of *S. aureus* FakB2 (1).

**ABJ55200.1_spFakB3 ---MTWKIIADSGCDYRQLPTPAINTTFVSVPLTIQV-ADQVFVDDASLD--IDQMMETM 54**

**WP_002357950.1_efFakB5 MNKEKIALLVDSGTDVPEALVKQYGMYVL--PLQIIY-PEKTYTDKVDIT--PEEVYQRL 55**

**WP_002359214.1_efFakB4 ---MNYQLVTDSCCDLPYTYLKENQVPFI--SMNIQI-DGKEYRDDLGETFDYQNFLTAI 54**

**ABD20605.1_saFakB1 ---MKIAVMTDSTSYLSQDLIDKYNIQIA--PLSVTFDDGKNFTESNEIA--IEEFYNKM 53**

**ABJ55113.1_spFakB1 ---MKLAVFTDSSAYLSAETLQREDLFVL--DIPVNI-DGEEYVEGINLS--AEEFYQKM 52**

**WP_002382324.1_efFakB1 ---MKIAIVTDSTAYLPERIKDHPNLFVI--PIPVIL-DGKIYNEGIDIE--ADEYYALL 52**

**ABD22554.1_saFakB2 --MTKQIIVTDSTSDLSKEYLEANNIHVI--PLSLTI-EGASYVDQVDIT--SEEFINHI 53**

**ABJ55472.1_spFakB2 --MTKIKIVTDSSVTIEPELVKQLDITIV--PLSVMI-DNVVYSDADLKE--EGKFLQLM 53**

**WP_002357444.1_efFakB2 --MTNVKIVTDSSCTMEKSLRDELNIHMM--PLSIMV-DGVVYPDDDHLP--GEKFMDMM 53**

**. :..** . : : : : : :**

**ABJ55200.1_spFakB3 YATAEASKSACPSPDDYLRAFE-----GAKNIFLVTITGTLSGSHNSAQLAKNIYLEDHP 109**

**WP_002357950.1_efFakB5 E--KEIPSTSLPDGATIQAIFDKIKEAGYEKVLAVTISSGLSGTYNVVRLLGEQ----TE 109**

**WP_002359214.1_efFakB4 KN-GSMPTTSQVNVGRYNEFFRPFVEQGL-PVIYLAFSSGLSGSYQSALQSVEMLKEEYD 112**

**ABD20605.1_saFakB1 ASSQTIPTTSQPAIGEWITKYEMLRDQGYTDIIVICLSSGISGSYQSSYQAGEM----VE 109**

**ABJ55113.1_spFakB1 AQASELPKTSQPSIAKLDEILTSLKEQGYTHALGLFLSSGISGFYQSIQYMVDD----YE 108**

**WP_002382324.1_efFakB1 NNSKEFPTTSQPALGEVLELYKSIAEQGYDTIISIHLSSGISGFVHTLHGLTDE----IP 108**

**ABD22554.1_saFakB2 ENDED-VKTSQPAIGEFISAYEELGKDGS-EIISIHLSSGLSGTYNTAYQASQM----VD 107**

**ABJ55472.1_spFakB2 QESKNLPKTSQPPVGVFAEIFEDLCKDGG-QILAIHMSHALSGTVEAARQGASL----ST 108**

**WP_002357444.1_efFakB2 ANAKALPKTSQPPIGEFVELYDRLGEDGS-EVISIHMTKGLSGTVEAARQASNL----SS 108**

**.:: * : : :: :** . .**

**ABJ55200.1_spFakB3 DTKIHVIDSLSAGGEVDLLVEKLNDLIDQGLSFEEVVEAITAYQEKTKLLFVLAKVDNLV 169**

**WP_002357950.1_efFakB5 GLDVFVLDTKNIGIGAGIQAIRAAELIETGLGWQELQQKLTEEVANAKVFFNVATLEYLQ 169**

**WP_002359214.1_efFakB4 NVEIHIIDTKAASLGQGMLVREAIRLQTDGHSLGEVVAYLEEQKMKLHSWVTVDDLKHLE 172**

**ABD20605.1_saFakB1 GVNVHAFDSKLAAMIEGCYVLRAIEMVEEGYEPQQIIDDLTNMREHTGAYLIVDDLKNLQ 169**

**ABJ55113.1_spFakB1 GLTIAFPDTLITSAPLGIMVESVFNWRDQGDDFASIQDKLAIQISRTSAFIMVDDLDHLV 168**

**WP_002382324.1_efFakB1 GVALYPYDSKITSMPMGHMVEAALDLTEEKASLEEIFAKLDLIRDNTYAYLIVEDLNNLV 168**

**ABD22554.1_saFakB2 -ANVTVIDSKSISFGLGYQIQHLVELVKEGVSTSEIVKKLNHLRENIKLFVVIGQLNQLI 166**

**ABJ55472.1_spFakB2 -ADVIVVDSSFTDQALKFQVVEAAKLAQEGKDMEAILSHVEEVKNHTELYIGVSTLENLV 167**

**WP_002357444.1_efFakB2 -SKVTVIDSDFTDQGLSFQVIQAAKLAQAGAGVPEILAEIERVKQNTKLYIGISTLDNLV 167**

**: *: : : . . : :. ***

**ABJ55200.1_spFakB3 KNGRLSKLIGTVVGLLNIRMVGKASETGTLELLQKARGSKKSVQAAYDELVKAG---YAG 226**

**WP_002357950.1_efFakB5 KGGRIGLVTSILGNALKLNPIISCNEEGIYYTVAKSRGRKKSLDKTFELVTNFIGEA-PR 228**

**WP_002359214.1_efFakB4 RGGRISKTAAALGGLMNIKPIIRVDAAGKLASVGKTRGRNKSLQKIAQETIQGIVEP-MK 231**

**ABD20605.1_saFakB1 KSGRITGAQAWVGTLLKMKPVLKFEDG-KIIPEEKVRTKKRAIQTLEKKVLDIVKDF-EE 227**

**ABJ55113.1_spFakB1 KGGRLSNGAAILGNLLSIKPILYFNDQGVIEVYEKVRTEKKATKRLIEIIKETTAS--GQ 226**

**WP_002382324.1_efFakB1 RGGRLTNGAALIAGLLKIKPILTFEDG-KIVLFEKIRSTKKAFARAEKIIGERNAGIEAP 227**

**ABD22554.1_saFakB2 KGGRISKTKGLIGNLMKIKPIGTLDDG-RLELVHNARTQNSSIQYLKKEIAEFIGDH-EI 224**

**ABJ55472.1_spFakB2 KGGRIGRVTGLLSSLLNIRVVMQMKDH-ELQPMVKGRGTKTFKKWLDELITSLSER--AV 224**

**WP_002357444.1_efFakB2 KGGRISRTTGLLSNIFNMKVVMDFENT-ELIPVAKGRGVKTFNKWFDELKSELSKIP-NV 225**

**:.**: . : :.:. : . : * : . .**

**ABJ55200.1_spFakB3 GRIVMAQRNNEKCCQQLSERIRETFPQ-ADIKILPTSGLCSFYAEEGGLLMGYEID---- 281**

**WP_002357950.1_efFakB5 FRLAVAHGAAEEEAKAMMERLKAAFPQAEEIYFGTISPALVVHTGPGLLGVGIQLLND-- 286**

**WP_002359214.1_efFakB4 QTLLIAYAGTKDDAEKVKELIEKEIEV-NEILIYPLGPTITSHTGIGCIAVFSFGEKRK- 289**

**ABD20605.1_saFakB1 VTLFVINGDHFEDGQALYKKLQDDCPSAYQVAYSEFGPVVAAHLGSGGLGLGYVGRKIRL 287**

**ABJ55113.1_spFakB1 YRVIVIHGNAPEKAEELRQHLLDFGLG-SDVSLATFGSVIGTHLGAGSIALGYIPVI--- 282**

**WP_002382324.1_efFakB1 VKLYVIHANNRIVAEKEQAKLQKLYPN-AEIEIGHFGPVIGTHLGEKAIGLAISAQ---- 282**

**ABD22554.1_saFakB2 KSIGVAHANVIEYVDKLKKVFNEAFHV-NNYDINVTTPVISAHTGQGAIGLVVLKK---- 279**

**ABJ55472.1_spFakB2 AEIGISYSGSDDWAKEMKESLQAY-VE-KPISVLETGSIIQTHTGENAWAILIRYHS--- 279**

**WP_002357444.1_efFakB2 RQIGISHADGLELANGFKEGLQAIFKD-MDIPVLHTNPVIATHTGKNAFAIMYYTD---- 280**

**: : . : : :**

**ABJ55200.1_spFakB3 - 281**

**WP_002357950.1_efFakB5 - 286**

**WP_002359214.1_efFakB4 - 289**

**ABD20605.1_saFakB1 T 288**

**ABJ55113.1_spFakB1 - 282**

**WP_002382324.1_efFakB1 - 282**

**ABD22554.1_saFakB2 - 279**

**ABJ55472.1_spFakB2 - 279**

**WP_002357444.1_efFakB2 - 280**

**Fig S2. Residues important for binding fatty acids are conserved in the *E. faecalis* FakB homologs.** Clustal Omega was used to align the protein sequences of saFakB1, saFakB2, spFakB1, spFakB2, spFakB3, efFakB1, efFakB2, efFakB4, and efFakB5. Residues necessary for Fak activity are highlighted (1). Red: residues are conserved with saFakB2; Blue: residues are similar to saFakB2. Note that spFakB3 had two substitutions for strongly similar amino acid residues at Thr-61 His-266 of saFakB2.


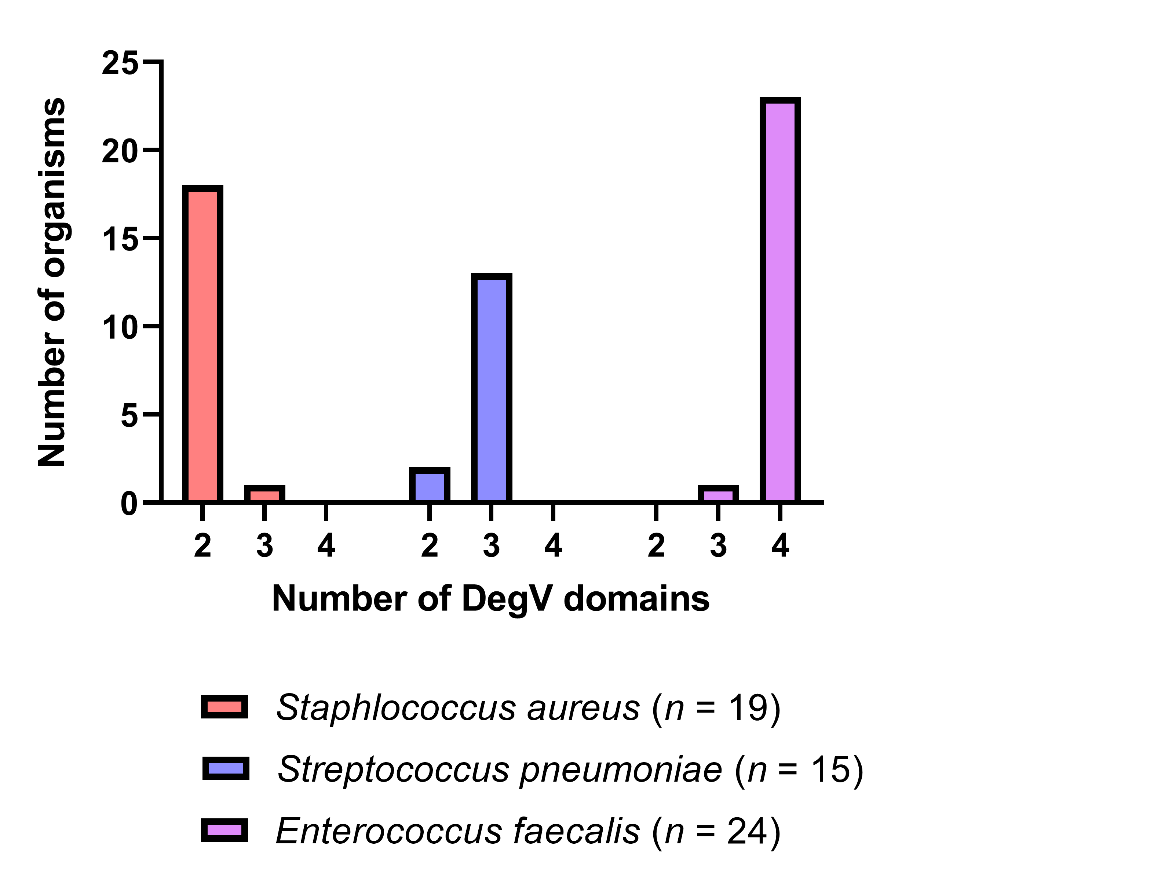


**Fig S3. DegV domain numbers of *Staphylococcus aureus*, *Streptococcus pneumoniae*, and *Enterococcus faecalis*.** Represented are the number of isolates of *Staphylococcus aureus* (red), *Streptococcus pneumoniae* (blue), and *Enterococcus faecalis* (purple) which had two, three, or four DegV domains.


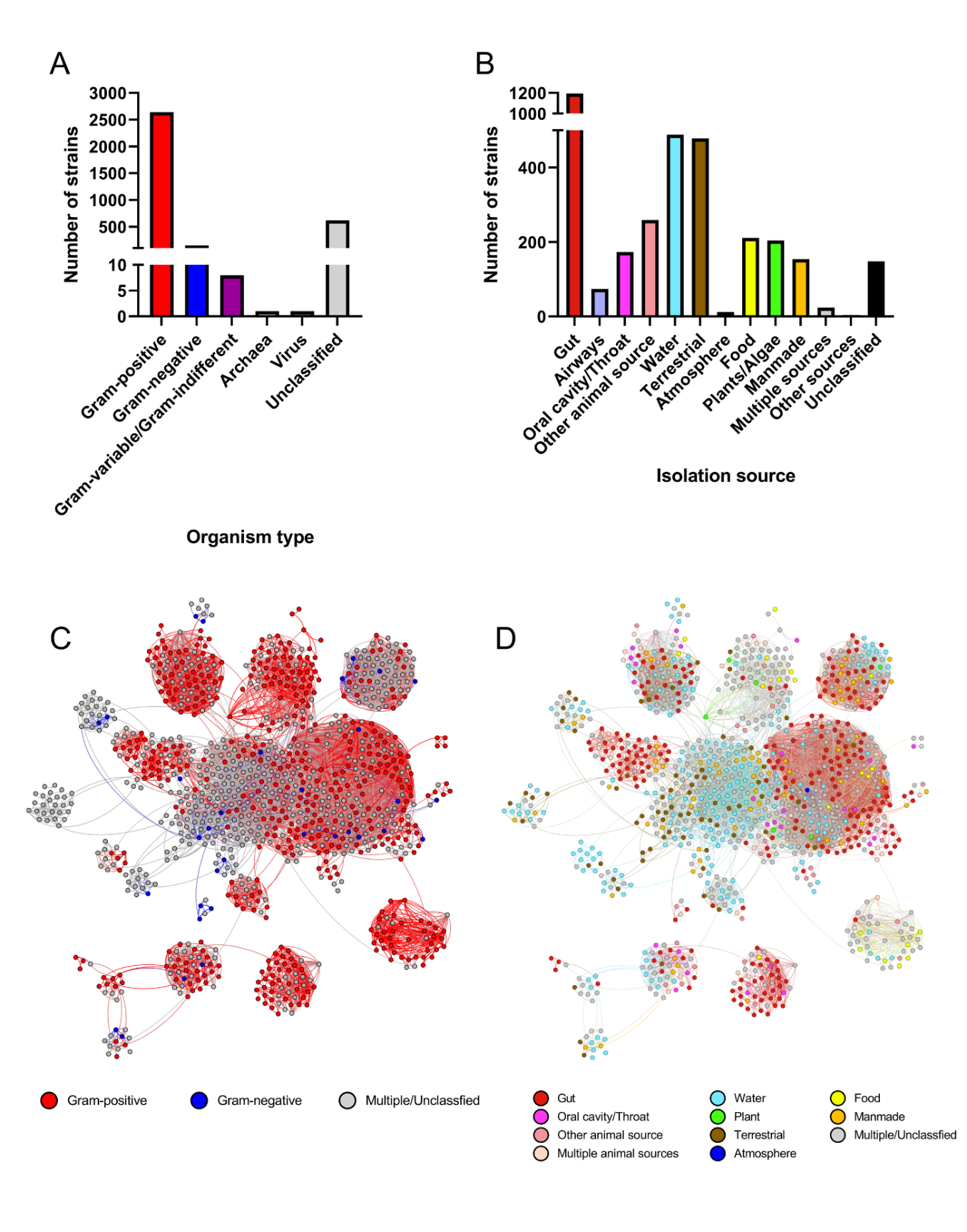


**Fig S4. Distribution of DegV network via species type and isolation source.**

(A) Number of organisms in the DegV network classified as Gram-positive, Gram-negative, Gram-variable or Gram-indifferent, archaea, virus, or organisms which could not be classified. (B) Number of organisms classified according to isolation source. In both A and B, shown are the raw numbers of organisms belonging to each classification. (C) Clustering of Gram-positive and Gram-negative organisms in the DegV network. (D) Clustering of organisms isolated from different sources in the DegV network.

**Fig S5**. Growth of *E. faecalis* OG1RF derivatives in TSB. Overnight cultures of the indicated strains were diluted to be 0.01 in TSB (tryptic soy broth) and growth monitored over time. Shown are the averages± standard deviations of *n*=3 cultures.


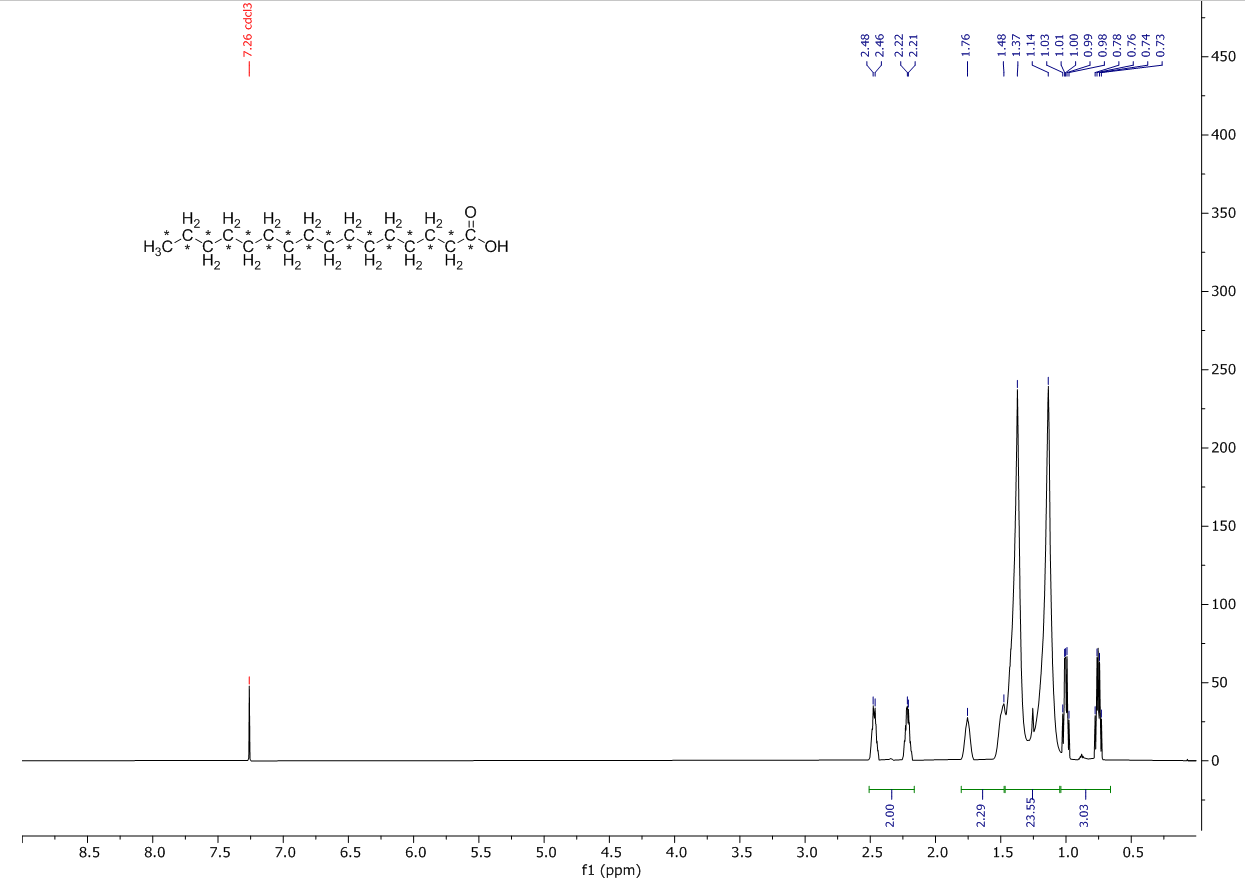


**Fig S6.** ^1^H NMR of Palmitic Acid (U-^13^C)


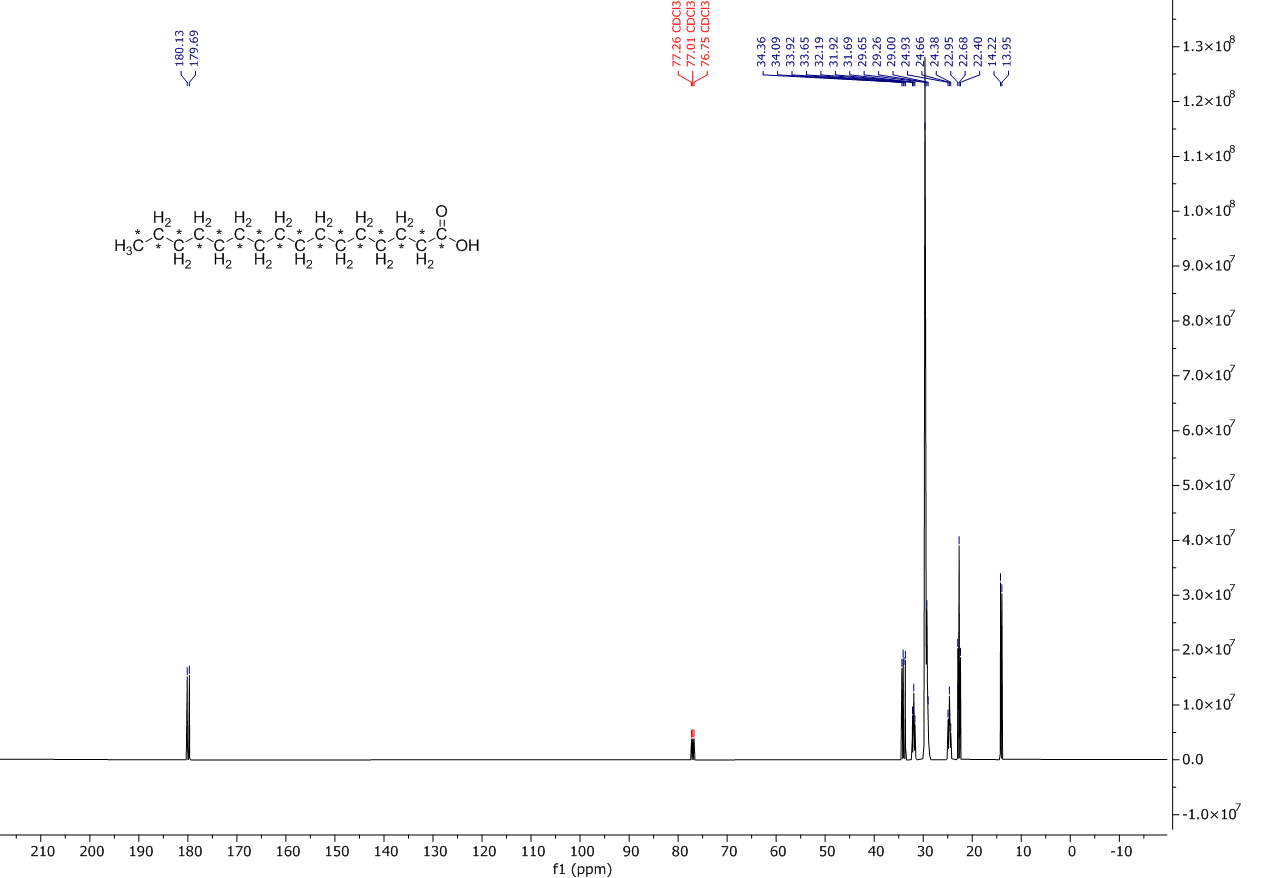


**Fig S7.** ^13^C NMR of Palmitic Acid (U-^13^C)

**REFERENCES**

1. **Broussard, T. C., D. J. Miller, P. Jackson, A. Nourse, S. W. White, and C. O. Rock.** 2016. Biochemical Roles for Conserved Residues in the Bacterial Fatty Acid-binding Protein Family. J Biol Chem **291:**6292-6303.

2. **Kristich, C. J., J. R. Chandler, and G. M. Dunny.** 2007. Development of a host-genotype-independent counterselectable marker and a high-frequency conjugative delivery system and their use in genetic analysis of *Enterococcus faecalis.* Plasmid **57:**131-144.

3. **Murray, B. E., K. V. Singh, R. P. Ross, J. D. Heath, G. M. Dunny, and G. M. Weinstock.** 1993. Generation of restriction map of *Enterococcus faecalis* OG1 and investigation of growth requirements and regions encoding biosynthetic function. J Bacteriol **175:**5216-5223.

4. **Saito, H. E., J. R. Harp, and E. M. Fozo.** 2018. *Enterococcus faecalis* Responds to Individual Exogenous Fatty Acids Independently of Their Degree of Saturation or Chain Length. Appl Environ Microbiol **84**.

5. **Saito, H. E., J. R. Harp, and E. M. Fozo.** 2014. Incorporation of exogenous fatty acids protects *Enterococcus faecalis* from membrane damaging agents. Appl Environ Microbiol.
